# Evaluating the impact of bioinspired counterion inclusion on silk nanoparticle physicochemical attributes and physical stability†

**DOI:** 10.1039/d5na00365b

**Published:** 2025-07-21

**Authors:** Napaporn Roamcharern, Panida Punnabhum, F. Philipp Seib, Zahra Rattray

**Affiliations:** a Strathclyde Institute of Pharmacy and Biomedical Sciences, University of Strathclyde 161 Cathedral St. Glasgow G4 0RE Scotland UK zahra.rattray@strath.ac.uk; b Fraunhofer Institute for Molecular Biology and Applied Ecology, Branch Bioresources Ohlebergsweg 12 35392 Giessen Germany; c Department of Pharmaceutical Technology and Biopharmaceutics, Institute of Pharmacy, Friedrich Schiller University Jena Lessingstr. 8 07743 Jena Germany

## Abstract

Silk fibroin is a promising material for nanocarrier-based drug delivery applications due to its biocompatibility, biodegradability, and mechanical properties, which can be fine-tuned through processing conditions. In this study, we explore the impact of Ca^2+^ and K^+^ inclusion on the morphology of silk nanoparticles and evaluate the short- and long-term stability of silk nanoparticles formed by antisolvent precipitation in deionized water and sodium phosphate buffer. Using advanced electric asymmetric flow field-flow fractionation multiplexed with online detectors (EAF4-UV-MALS-DLS) and orthogonal analytics (DLS, ELS, NTA, FE-SEM), we analyze the physicochemical attributes of silk nanoparticles. We find significant differences in nanoparticle architecture and stability in different buffers, with notable differences in particle size (*R*_g_ and *R*_h_), charge, and shape measured over 56 days. Notably, nanoparticles formulated with 0.7 mg Ca^2+^ and 1.1 mg K^+^ maintained superior physicochemical stability, making them promising candidates for future nanocarrier-based applications.

## Abstract 2

Silk fibroin is a promising material for nanocarrier-based drug delivery applications due to its biocompatibility, biodegradability, and mechanical properties, which can be fine-tuned through processing conditions.

## Graphical abstract

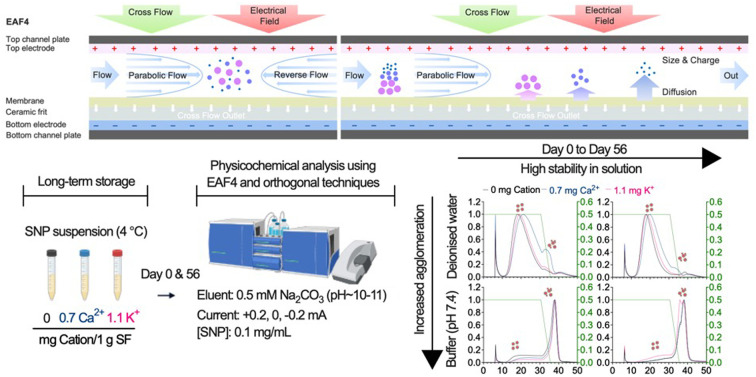

## Introduction

Nano-sized drug delivery systems have gained substantial attention as vehicles for improving targeted bioactive drug delivery and the controlled release of biomedical and pharmaceutical therapies.^1^ Bioinspired protein-based nanocarriers offer distinct advantages for nanoscale drug delivery applications.^2^ For example, silk fibroin from *Bombyx mori* stands out due to its favorable physicochemical properties, including mechanical strength,^3^ biocompatibility,^4^ biodegradability,^4^ and broad-spectrum applications in nanostructured biomaterials processing for nanomedicine.^5^

The self-assembly of silk fibroin and the thermodynamic processes that regulate it have been a focus of past research. Emerging evidence confirms that this assembly is influenced by the molecular mobility, charge, hydrophilic interactions, and concentration of silk fibroin.^6^ Thus, modifying the physicochemical properties of silk fibroin can give rise to novel nanostructure architectures and conformations, thereby enhancing the manufacturability of silk nanoparticles.

Silk nanoparticles can be generated using various bottom-up protocols, including desolvation, coacervation, emulsification, antisolvent precipitation, nano spray drying, and electrospraying.^2^ Antisolvent precipitation involves the rapid desolvation of soluble silk proteins in a solvent to form protein precipitates that can entrap drug cargo.^7^ The physicochemical attributes of the resulting nanoparticles are influenced by the choice of protocol parameters, including organic solvent composition, volume ratio of non-solvent to organic solvent, injection speed, and stirring rate.^7,8^ In a previous study, we successfully manufactured silk nanoparticles using a antisolvent precipitation method in a semi-batch format that included the incorporation of Ca^2+^ and K^+^ cations to improve silk nanoparticle production yield. This method resulted in a range of critical quality attributes, with a diameter in the 93–290 nm range, a polydispersity index <0.15, and zeta potential in the −39 to −52 mV range.^9^ These properties of silk nanoparticles, together with their negligible cytotoxicity and undetectable induction of inflammatory responses in macrophages, suggested that silk nanoparticles are promising for biomedical and pharmaceutical applications. Our further FTIR and NMR studies of the impact of Ca^2+^ on silk fibroin solution-phase properties demonstrated structural transitions from liquid silk (silk I) to solid silk nanoparticles (silk II).^9^

Despite their potential, nanoparticles for therapeutic applications face numerous translational challenges related to their physical properties, such as colloid stability, swelling behavior, and crystallinity.^10^ Loss of physical stability, particularly colloidal stability, negatively impacts the biological fate of protein-based nanocarriers *in vivo* by triggering immunogenicity and reducing circulation time.^11,12^ Thus, early development efforts required comprehensive analysis of critical nanoparticle quality attributes. Previous work demonstrated that silk nanoparticles and other silk fibroin-capped nanoparticles possess enhanced colloidal stability across broad ranges of pH and ionic strength due to steric hindrance effects. Moreover, the surface modification of silk nanoparticles with cationic polymers improved their colloidal stability in biological media.^13,14^

However, the effect of counterion inclusion on the physicochemical stability of silk nanoparticles remains understudied. Studies on potassium chloride-loaded alginate beads, a model study for counterion interaction showed that calcium promotes stable crosslinking, while potassium affects entrapment and gel structure.^15^ These findings highlight the importance of ion interactions and the need to explore their role in silk nanoparticle stability.

The observation of a significant impact of cations on silk structure during silk nanoparticle formation led us to speculate that cation-induced structural changes may directly influence the functionality and stability of silk. Due to a paucity of reports on the long-term stability of silk nanoparticles in the absence of stabilizing agent—despite some evidence on short-term stability (∼24 h)^16^—we conducted further investigations into the physical stability of cation-mixed silk nanoparticles using electrical asymmetrical flow-field flow fractionation (EAF4), a novel separation based approach that has gained attention in the nanoscale separation and analysis in recent years. AF4 is a subtechnique of flow field-flow fractionation (FFF) and involves the application of a hydrodynamic force (cross flow) to the elution flow. Under parabolic flow in the FFF channel, analyte velocities vary based on their unique physicochemical properties, leading to effective fractionation with different elution times.^17–20^ EAF4 incorporates an additional electrical field from electrodes to enhance the separation of charged analytes with different electrophoretic mobilities and diffusion coefficients.^19^

In the present study, we provide the first report of the physical stability of silk nanoparticles prepared by antisolvent precipitation in the presence of Ca^2+^ and K^+^ cations. Using an EAF4-multidetector setup and orthogonal analytical techniques, we report the short-term and long-term storage stability of these silk nanoparticles and provide new insights into their physicochemical properties.

## Experimental

### Aqueous silk fibroin preparation

Silk fibroin solution was prepared from *Bombyx mori* cocoons by degumming and dissolution, using a method described elsewhere.^19^ For the purpose of this article, we use the term “silk” to refer to silk fibroin.

Dried degummed silk was dissolved in LiBr at 60 °C for 4 h under gentle stirring, and subsequently dialyzed against deionized water using a 3.5 K MWCO dialysis cassette (Slide-A-Lyzer 3.5K Dialysis Cassette G2, Thermo Scientific, Rockford, IL, USA) for 48 h at ambient temperature. The silk solution was collected following centrifugation (2885×*g*, 40 min at 4 °C) (PK 121R Centrifuge, rotor T515, ACL International Srl, Milan, Italy). Silk concentration (% w/v) was calculated from the dried weight of silk obtained by incubating 500 µL aliquots at 60 °C for 24 h.

### Silk nanoparticle manufacture

Cation-formulated silk nanoparticles were assembled following an antisolvent precipitation method reported elsewhere.^21^ Briefly, a cation-mixed silk solution was prepared to yield 3% w/v silk supplemented with CaCl_2_ (Ca^2+^ : silk; 0.7 : 1 and 11.5 : 1 mg/g) and KCl (K^+^ : silk; 1.1 : 1 and 17.3 : 1 mg/g) as these concentrations exhibited optimal physicochemical properties,^9,22^ and closely reflect the mass ratio in the silkworm middle silk gland.^23–25^ Then, 6 mL of the mixture was dropped from a height of 7.5 cm above the meniscus into 30 mL of isopropanol at a syringe pump speed of 1 mL/min and a stirring rate of 400 rpm. The silk nanoparticles were centrifuged at 48400×*g* for 2 h at 4 °C, and sonicated twice at 30% amplitude for 30 s in deionized water. This washing and centrifugation cycle was repeated twice more. The silk nanoparticles were manufactured from three independent silk batches.

### Temporal physical stability evaluation of silk nanoparticles

**Fig. 1:**
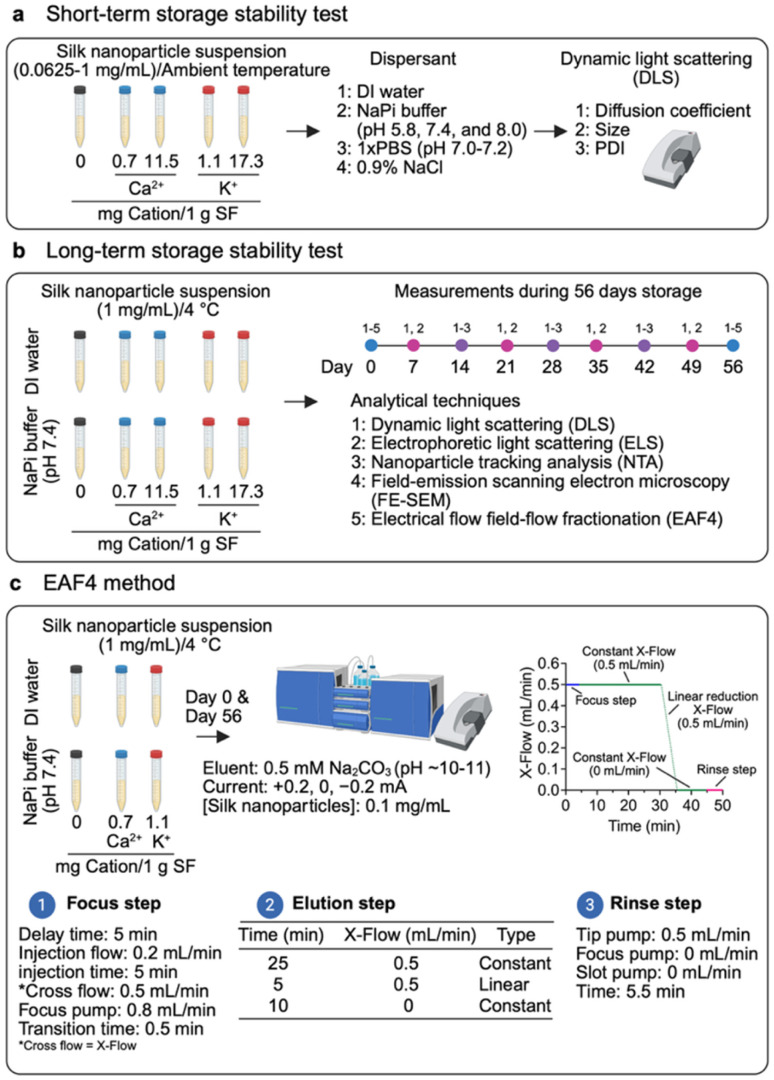
Schematic of silk nanoparticle storage stability study illustrating measurement workflow and analytical techniques, performed (a) after 2 h incubation at ambient temperature, (b) over 56 days, and (c) the electrical asymmetric flow field-flow fractionation (EAF4) method used for nanoparticle characterization at day 0 and day 56. Abbreviations: deionized water (DI water); sodium phosphate buffer (NaPi buffer).

Short-term colloidal stability of silk nanoparticles was evaluated prior to long-term storage (56 days) by storing silk nanoparticles in the 0.0625–1 mg/mL concentration range, dispersed in deionized water, sodium phosphate buffer (10 mM, pH 7.4), 1× phosphate-buffered saline (pH 7.0–7.2) (PBS, 137 mM NaCl, 2.7 mM KCl, 10 mM Na_2_HPO_4_, and 1.8 mM KH_2_PO_4_−pH 7.0–7.2), and 0.9% w/v NaCl (normal saline). The impact of pH (pH 5.8, 7.4, and 8.0) was also assessed in 10 mM sodium phosphate buffer. Diffusion coefficient, particle size, and polydispersity index (PDI) were measured to evaluate physical (colloidal) stability under each condition by dynamic light scattering (DLS), following a 2 h incubation at ambient temperature (Fig. 1a).

For long-term stability, the stability of silk nanoparticle suspensions was determined in deionized water and 10 mM sodium phosphate buffer (pH 7.4). Aliquots of 1 mg/mL silk nanoparticle suspension were prepared in each solution at 4 °C. Their physicochemical properties were tracked by dynamic light scattering (DLS), electrophoretic light scattering (ELS), nanoparticle tracking analysis (NTA), asymmetric electrical flow field-flow fractionation (EAF4), and field emission scanning electron microscopy (FE-SEM). The analytical workflow, including sampling timepoints, is presented in Fig. 1b.

### Particle size and zeta potential determination

Particle size (Z-average) and PDI were measured using a Zetasizer Nano ZS (Malvern Panalytical, Worcestershire, UK) with a 633 nm helium–neon laser and a 173° detection angle (non-invasive back scattering setting). All measurements were performed using a 1 : 25 ratio (v/v) of silk nanoparticles: (i) 0.0625–1 mg/mL for short-term storage and (ii) 1 mg/mL for long-term storage dispersed in deionized water, with a count rate of 203.6 kcps, a measurement duration of 70 s, a measurement position at 3 mm, and measurement temperature at 25 °C. The diffusion self-interaction parameter, *k*_D_, was derived from the slope of the diffusion coefficient of silk nanoparticle samples as a function of concentration (0.0625–1 mg/mL).

Zeta potential was measured using electrophoretic light scattering (Zetasizer Nano-ZS Malvern Instrument) and a 1 : 20 ratio (v/v) of long-term storage silk nanoparticle suspension (1 mg/mL) to deionized water at 25 °C, a count rate of 161.5 kcps, and a measurement position of 2 mm. Each silk nanoparticle type was analyzed as three independent replicates consisting of five technical replicates for DLS and three technical replicates for ELS.

### Nanoparticle tracking analysis (NTA)

At each stability time point (Fig. 1b), the silk nanoparticle suspension (1 mg/mL) was aliquoted, and diluted in Milli-Q water to achieve 20–80 particles per frame for NTA data acquisition. The corresponding particle size and concentration were measured alternate weekly using Nanoparticle Tracking Analysis (NTA 3.4 v3.4.003, Malvern Panalytical Ltd, Worcestershire, UK) configured with a sCMOS camera, a 488 nm laser, and a syringe pump set at 100 (Harvard Apparatus Model 98-4730 Syringe Pump, Massachusetts, US).

Measurements were acquired at ambient temperature and recorded for five replicate videos of 60 s duration. Capture settings included a camera level of 9 for the smaller-sized silk nanoparticle samples (0 mg cation, 0.7 mg Ca^2+^, and 1.1 mg K^+^) and a camera level of 8 for the larger nanoparticle samples (11.5 mg Ca^2+^ and 17.3 mg K^+^). Data were processed using NTA 3.4 (version 3.4.003) software with a detection threshold of 6.^26^ Each silk nanoparticle type was analyzed as three independent replicates consisting of five technical replicates.

### Analysis of silk nanoparticle morphology and size by field emission scanning electron microscopy (FE-SEM)

Silk nanoparticle suspensions were aliquoted on days 0 and 56 and gently vortexed to achieve resuspension. A droplet of nanoparticle suspension was transferred onto a 5 × 5 mm silicon wafer chip (Ted Pella, Inc., CA, USA), dried at ambient temperature for 24 h, and sputter-coated with gold from a height of 35 mm for 40 s at 0.08 mb and 30 mA (Agar Scientific Manual Sputter Coater, Agar Scientific Ltd, Essex, UK). The silk nanoparticles were imaged by FE-SEM at 20 000× and 60 000× magnifications at 5 kV (Hitachi SU6600, Hitachi High-Tech Europe GmbH, Krefeld, Germany).

### Analysis of silk nanoparticle physical stability by multidetector electrical asymmetric flow field-flow fractionation (EAF4)

Silk nanoparticles were separated, and particle stability was tested at days 0 and 56 using an EAF2000 Asymmetrical Flow FFF system (AF2000, PostNova Analytics, Landsberg am Lech, Germany), controlled by an electrical module (#PN2411, Postnova Analytics, Landsberg am Lech, Germany), and equipped with a conductivity meter. The spacer thickness was 350 µm, and a regenerated cellulose membrane (modified RC membrane) with a molecular weight cut-off of 10 kDa (Postnova AF2000 MF-AF4 Analytic RC Membrane, Z-AF4-MEM-618-10 kDa, Postnova Analytics, Landsberg am Lech, Germany) was used as the accumulation wall for all measurements. The in-line detectors multiplexed with EAF4 were a 21-multi-angle light scattering (MALS) detector (#PN3621, Postnova Analytics), a refractive index (RI) detector (PN3150, Postnova Analytics), a UV detector (#SPD-M40, Postnova Analytics), and an online Zetasizer Nano ZS (Malvern Panalytical).

EAF4 separation and inline analysis were performed on days 0 and 56 for silk nanoparticles suspended in deionized water (0.1 mg/mL). The EAF4 method used is described elsewhere^27^ with minor modifications that included an injection volume of 20 µL, a 0.5 mM Na_2_CO_3_ (∼pH 10) carrier solution (0.1 µm pore size filtered), and an applied current of ±0.2 mA (Fig. 1c). The term “AF4” used in this study, from this point onward, refers to EAF4 data analyzed under a neutral current (0 mA) only.

Nanoparticle shape factor was determined according to the radius of gyration (*R*_g_) derived from MALS and the hydrodynamic radius (*R*_h_) from inline DLS measurements eqn (1).1
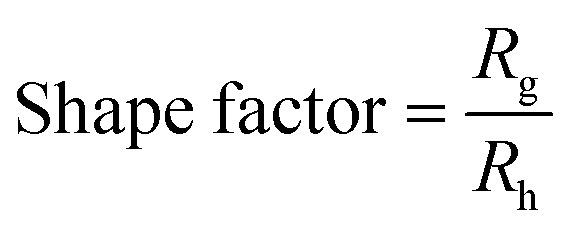


Electrophoretic mobility (*µ*) and zeta potential (*ζ*) were evaluated using the Nova FFF-EAF4 Software Module (Postnova Analytics). The *µ* (mm cm V^−1^ s^−1^) was calculated as a function of the correlation between the drift velocity induced by the electrical field (*n*_em_) and electrical field strength (*E*) using eqn (2–4).2
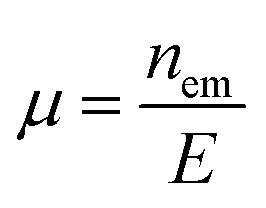

3 *n*_em_ = *n* − *n*_c_

where *n* is the total drift velocity and *n*_c_ the drift velocity induced by the cross flow.4
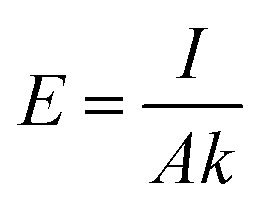
where *I* is the applied current, *A* is the channel area, and *k* is the conductivity.

Zeta potential (mV) was estimated using an input parameter of standard nanoparticles with Smoluchowski approximation *f*(*ka*) of 1.5 (eqn (5)).5
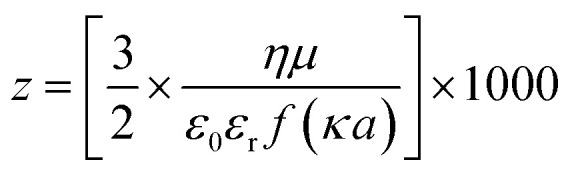
where *η* is viscosity at 25 °C of eluent (Pa s), *ε*_0_ the vacuum permittivity (physical constant) (∼8.854 × 10^−12^ As V^−1^ m^−1^), *ε*_r_ is the relative permittivity of water at 25 °C (∼78.53114), *µ* is calculated electrophoretic mobility (mm cm V^−1^ s^−1^), *f*(*ka*) is Henry's function, *k* the reciprocal of Debye length, *R*_h_ the hydrodynamic radius, and *ka* the estimated ratio of the particle radius to the electrical double layer.

### Statistical analyses and software

GraphPad Prism 9 (GraphPad Software, Boston, MA, USA) was used for the graphs and statistical analyses, including two-way ANOVA, Dunnett's multiple comparisons test, and Šídák's multiple comparisons test. Asterisks denote statistical significance, experimental repeats (*n*) are specified in each figure legend, and all data are presented as mean values ± (SD). The cartoon illustration was prepared using Microsoft PowerPoint version 16.93 (Microsoft^®^ PowerPoint for Mac, Redmond, WA, USA) and BioRender (Scientific Image and Illustration Software, https://www.biorender.com). All AF4 and EAF4 data were analyzed using NovaFFF AF2000 Software (Postnova Analytics).

## Results and discussion

### Physical stability of silk nanoparticles

**Fig. 2:**
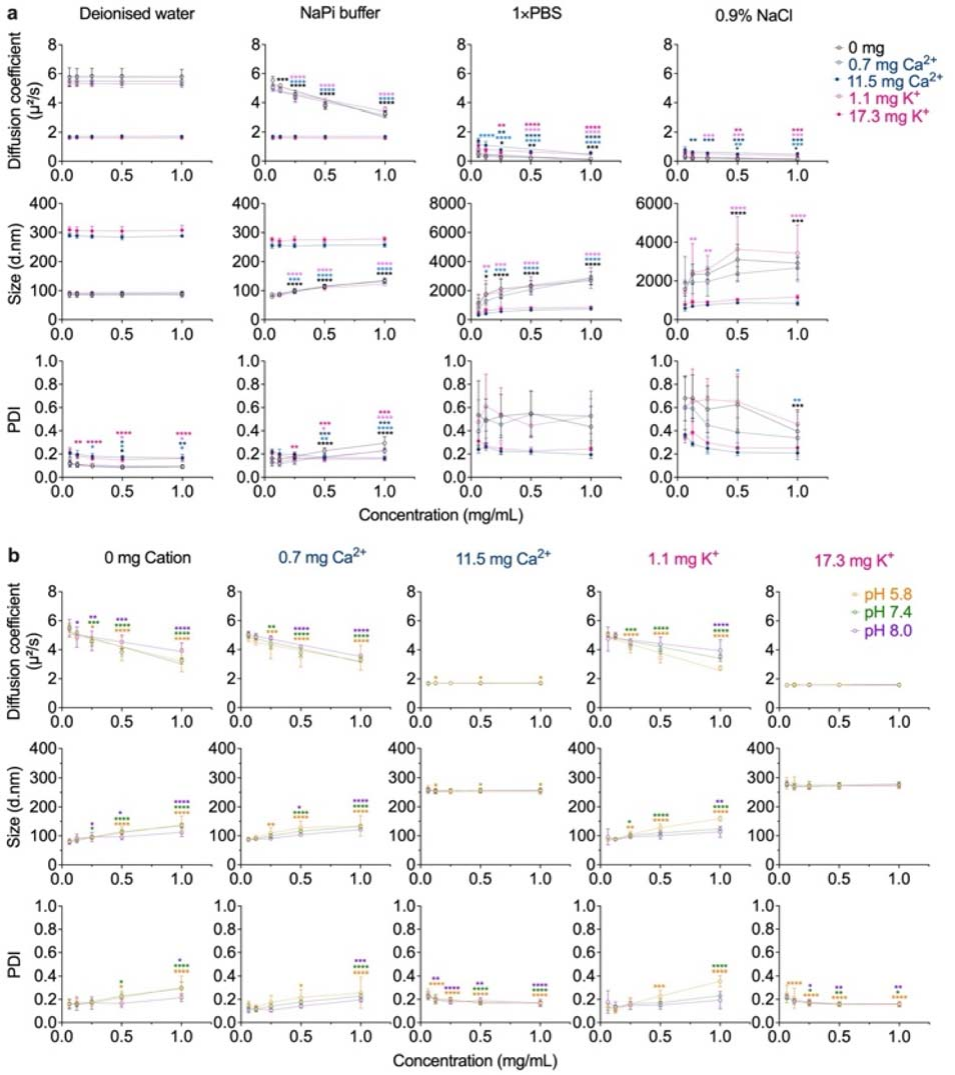
Colloidal stability of silk nanoparticles. Diffusion coefficients, particle size, and polydispersity index (PDI) were determined by dynamic light scattering (DLS) as a function of nanoparticle concentration. (a) The impact of continuous phase, which was either deionized water (DI water), 10 mM sodium phosphate (NaPi) buffer (pH 7.4), 1× phosphate-buffered saline (PBS) (pH 7.0–7.2), or 0.9% w/v NaCl, on nanoparticle agglomeration behavior (*n* = 3). (b) Agglomeration behavior of silk nanoparticles as a function of nanoparticle concentration and pH (5.8–8.0) was assessed in 10 mM NaPi buffer (*n* = 3). All conditions were measured after 2 hour incubation at ambient temperature. Two-way ANOVA and Dunnett's multiple comparisons test were used in the statistical analysis, using 0.0625 mg/mL as a control baseline: *p* < 0.05 (*), *p* < 0.01 (**), *p* < 0.001 (***), and *p* < 0.0001 (****). Colored asterisks represent statistical analyses for each sample and condition; 0 mg cation (control, black), 0.7 mg Ca^2+^ (light blue), 11.5 mg Ca^2+^ (blue), 1.1 mg K^+^ (light pink), 17.3 mg K^+^ (pink), pH 5.8 (orange), pH 7.4 (green), pH 8.0 (purple).

The colloidal stability of the silk nanoparticles was investigated as a function of concentration, formulation buffer composition, and pH, following a 2 hour incubation at ambient temperature (Fig. 2). Silk nanoparticles exhibited high physical stability in deionized water at 2 h, with minimal alterations in the diffusion coefficient, particle size, and PDI. Only minor changes in size were observed for nanoparticles dispersed in sodium phosphate buffer (10 mM, pH 7.4). However, the particle diameter increased over the tested concentration range. In the absence of cations, silk nanoparticles were 79–135 nm in diameter, while nanoparticles prepared with 0.7 mg Ca^2+^ were in the 87–132 nm size range, and those formulated with 1.1 mg K^+^ ranged from 86–123 nm in diameter. In contrast, silk nanoparticles formulated with higher amounts of Ca^2+^ (11.5 mg) and K^+^ (17.3 mg) were approximately 255 nm and 276 nm in diameter, respectively, and were relatively stable, exhibiting only minimal changes in particle diameter. Increases in particle diameter and PDI changes were also seen for nanoparticles dispersed in phosphate-buffered saline (pH 7.0–7.2) and 0.9% w/v NaCl, resulting in micron-scale particle complex formation (up to ∼3.6 d.µm) (Fig. 2a). Under different pH conditions (pH 5.8, 7.4, and 8.0), particle growth was observed—specifically for the low mass ratio ions, with an increase in particle size and PDI within a 2 h incubation time. In contrast, no significant difference was observed in particle growth for silk nanoparticles formulated with high mass ratio ions, and negligible changes in PDI were noted (Fig. 2b).

The diffusion self-interaction parameter, *k*_D_, can be used to estimate particle interaction behavior in a particular dispersant as a predictor of physicochemical stability. A negative *k*_D_ value indicates a slow diffusion rate due to aggregation or clustering (attractive force), while a positive *k*_D_ value indicates a higher diffusion rate, which causes a greater dispersity of particles (repulsive force).^28,29^ Therefore, the increased particle size with a less negative *k*_D_ produced in PBS and normal saline indicated silk nanoparticle aggregation (Table 1). A possible explanation for these observations is the salting-out effect at higher ionic strength—resulting from the presence of monovalent and divalent cations, including Ca^2+^ and K^+^, as well as phosphate salts in the buffer. We hypothesized that these components influence surface charge shielding and modulate the electrical double layer,^30,31^ thereby reducing electrostatic repulsion between particles, which leads to decreased solubility and increased aggregation.^32,33^

**Table 1:** Measured diffusion self-interaction parameter (*k*_D_) of silk nanoparticles in different dispersants (*n* = 3). Abbreviations: deionized water (DI water); phosphate buffered saline (PBS); sodium phosphate buffer (NaPi buffer)

*k*_D_ (mL/mg)						
Cation	DI water	NaPi buffer (10 mM)			1× PBS (pH 7.0–7.2)	NaCl (0.9% w/v)
		pH 5.8	pH 7.4	pH 8.0		
0 mg cation	−0.014	−2.063	−2.373	−1.335	−0.237	−0.103
0.7 mg Ca^2+^	−0.030	−1.467	−1.819	−1.592	−0.440	−0.155
11.5 mg Ca^2+^	0.010	0.047	−0.010	−0.006	−0.714	−0.206
1.1 mg K^+^	−0.065	−2.442	−1.597	−0.985	−0.389	−0.220
17.3 mg K^+^	0.001	0.044	−0.025	−0.002	−0.414	−0.176

### Physicochemical characteristics of silk nanoparticles in deionized water and sodium phosphate buffer

Beyond the ionic strength of the continuous phase, pH plays an important role in colloidal stability impacting the protonation and deprotonation state of charged solvent-exposed amino acid residues, silk protein structures, and conformational stability.^32–34^ Therefore, we also examined silk nanoparticle colloidal stability at pH 5.8–8.0 in sodium phosphate buffer (Fig. 2b and Table 1). The pH of the extracellular (pH 6–6.5) and endo-lysosomal (pH 4.5) microenvironments of cancer cells,^35^ may influence the performance and effectiveness of nanocarriers. Therefore, reduced particle stability (*e.g.*, agglomeration) under these pH conditions could adversely impact both cellular and intracellular delivery,^36,37^ increase the risk of cytotoxicity to normal cells,^38^ and delay the release of encapsulated drug cargoes.^38^

**Fig. 3:**
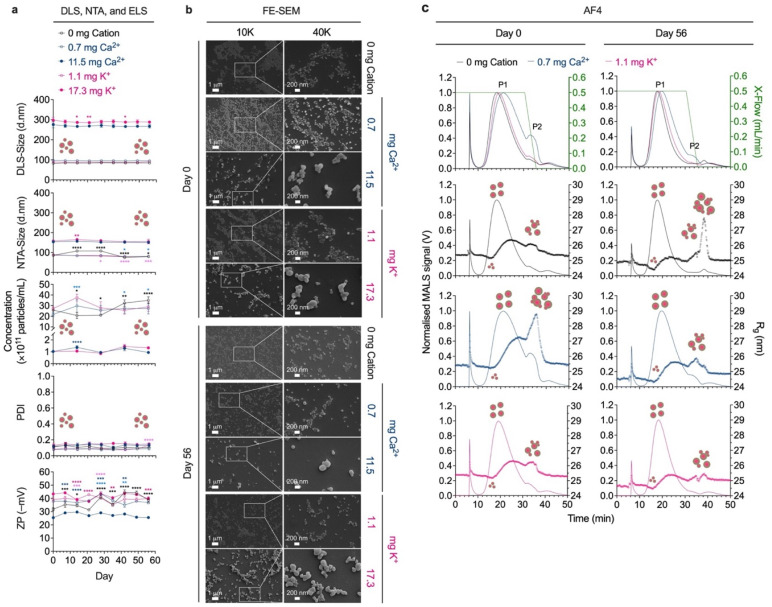
Storage stability of silk nanoparticles in deionized water at 4 °C. (a) Particle size, concentration, polydispersity index (PDI), and zeta potential (ZP) measured over 56 storage days in deionized (DI) water at 4 °C (*n* = 3). (b) SEM images (10 000× and 40 000× magnifications) of silk nanoparticles dispersed in deionized water at 4 °C on day 0 and day 56 (*n* = 1). (c) The fractograms of silk nanoparticles obtained from the conventional asymmetric flow field-flow fractionation (AF4) (no applied current; 0 mA) (*n* = 3). Two-way ANOVA and Dunnett's multiple comparisons test were used in statistical analysis, comparing the impact of storage day to day 0; *p* < 0.05 (*), *p* < 0.01 (**), *p* < 0.001 (***), and *p* < 0.0001 (****). Different colored asterisks represent statistical analysis for each sample; 0 mg (control, black trace), 0.7 mg Ca^2+^ (light blue trace), 11.5 mg Ca^2+^ (blue trace), 1.1 mg K^+^ (light pink trace), 17.3 mg K^+^ (pink trace). Abbreviations: dynamic light scatter (DLS); nanoparticle tracking analysis (NTA); electrophoresis light scattering (ELS); field-emission scanning electron microscopy (FE-SEM); multiangle light scatter (MALS); radius of gyration (*R*_g_); crossflow (X-flow).

At physiological pH (pH 7.4), silk nanoparticles exhibited different agglomeration behaviors, with 0 mg cation nanoparticles forming large agglomerates (lowest negative *k*_D_ value). Nanoparticles formulated in the presence of 0.7 mg Ca^2+^ and 1.1 mg K^+^ also formed larger aggregates in comparison to nanoparticles formulated with 11.5 mg Ca^2+^ and 17.3 mg K^+^ that were stable (Fig. 2b and Table 1). These observations highlight key factors involved in silk nanoparticle agglomeration, such as surface charge and particle concentration. At a constant protein concentration, the number of smaller nanoparticles was higher than that of larger nanoparticles (Fig. 3a). This increase in particle number leads to greater particle interactions and collisions,^39,40^ promoting agglomerate growth. As a result, the smaller particles exhibited a higher aggregation rate.

Negligible changes in agglomeration were observed in response to pH among the silk nanoparticles; however, the extent of particle agglomeration was more pronounced at pH 5.8 in comparison to pH 8.0 conditions. The theoretical isoelectric point (pI) for the silk H-chain is 4.39;^41^ thus, low pH conditions would induce less deprotonation, resulting in a lower net negative charge. This trend has been reported elsewhere, demonstrating that acidic conditions can modulate aggregate size and increase the volume of silk protein.^42,43^ Furthermore, silk nanoparticles exhibited a strong negative zeta potential of approximately −45 mV. We propose that an increased protonated state of the NH_2_ group and/or destabilization of the counter ions (Ca^2+^/K^+^) could explain this observation, as this would lead to a reduced particle net surface charge (less negative) and alter the protein conformation/particle shape, resulting in reduced interparticle repulsive interactions to drive particle agglomeration.^34^ However, silk nanoparticles formulated with counter ions (Ca^2+^/K^+^) ameliorate particle agglomeration comparable to control silk nanoparticles (0 mg cation).

Silk nanoparticles act in a similar manner to a charge-shifting nanoparticle, offering a promising strategy for designing pH-responsive silk nanoparticles for targeted drug delivery with tunable cellular uptake (100–200 nm diameter)^44^ and controlled release properties.^45^ Proof of principle comes from control silk nanoparticles that were demonstrated to exhibit pH responsive drug release,^46^ charge dependent silk–drug interactions,^47^ and lysosomotropic drug delivery in single live cells.^48^

**Fig. 4:**
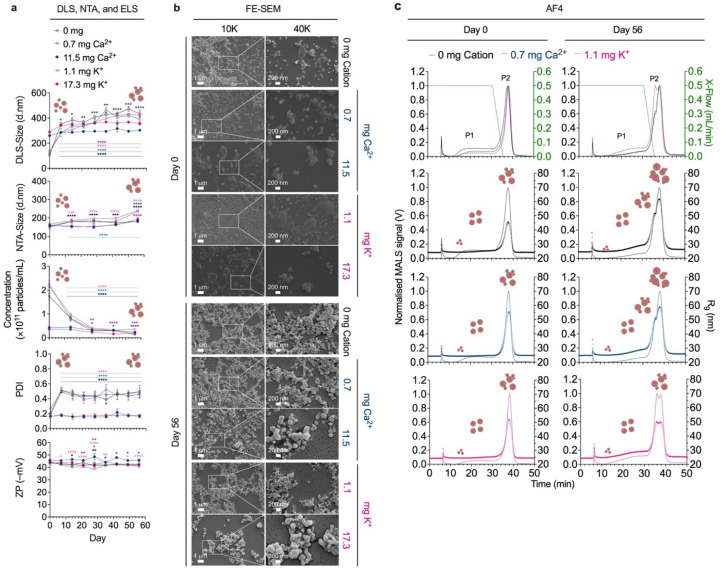
Storage stability of silk nanoparticles in sodium phosphate (NaPi) buffer (pH 7.4) at 4 °C. (a) Particle size, concentration, polydispersity index (PDI), and zeta potential (ZP) measured over 56 storage days in 10 mM NaPi buffer (pH 7.4) at 4 °C (*n* = 3). (b) FE-SEM micrographs (10 000× and 40 000× magnifications) of silk nanoparticles dispersed in 10 mM NaPi buffer (pH 7.4) at 4 °C on day 0 and day 56 (*n* = 1). (c) Corresponding fractograms of nanoparticles obtained from conventional asymmetric flow field-flow fractionation (AF4) (*n* = 3). Two-way ANOVA and Dunnett's multiple comparisons test were used in statistical analysis, comparing the impact of storage duration; *p* < 0.05 (*), *p* < 0.01 (**), *p* < 0.001 (***), and *p* < 0.0001 (****). Different color of an asterisk represents statistical analysis for each sample; black: 0 mg cation, light blue: 0.7 mg Ca^2+^, blue: 11.5 mg Ca^2+^, light pink: 1.1 mg K^+^, pink: 17.3 mg K^+^. Abbreviations: dynamic light scattering (DLS); nanoparticle tracking analysis (NTA); electrophoretic light scattering (ELS); field-emission scanning electron microscopy (FE-SEM); multiangle light scattering (MALS); radius of gyration (*R*_g_); crossflow (X-flow).

Silk nanoparticle stability at 4 °C in deionized water and sodium phosphate buffer was studied using multiple orthogonal analytical techniques (Fig. 3 and 4). Nanoparticle suspension stability was initially measured using DLS and NTA, which demonstrated no significant changes in measured particle size or PDI over 56 days of storage in deionized water—especially for smaller particles (Fig. 3a). In contrast, significant agglomeration was observed for silk nanoparticles dispersed in sodium phosphate buffer, particularly in the case of nanoparticles with a smaller baseline particle size (*i.e.*, 0 mg cation, 0.7 mg Ca^2+^, and 1.1 mg K^+^), which showed a 3- to 4-fold increase in particle size at day 56. A 6- to 12-fold decrease in particle concentration was measured for nanoparticles dispersed in sodium phosphate buffer (pH 7.4) over 56 days of storage. Negligible changes in nanoparticle surface charge were observed, demonstrating stable zeta potential (ZP) throughout storage (Fig. 3a and 4a). Observations from FE-SEM were consistent with the DLS and NTA size data and confirmed the formation of nanoparticle agglomerates after 56 days of storage at 4 °C in sodium phosphate buffer, which were absent for samples stored in deionized water (Fig. 3b and 4b). The impact of temperature on silk nanoparticle stability was profiled at ambient (25 °C) and physiologically relevant temperatures (37 °C), showing that storage at 37 °C for control and 17.3 mg K^+^ nanoparticles, resulted in the formation of micron-sized silk nanoparticle agglomerates (∼5 µm) (Fig. S1†). In addition, we also conducted a preliminary study in conditions mimicking cell culture, DMEM supplemented with 10% FBS, following 2 and 24 h incubation at 37 °C. Our results demonstrate changes in the nanoparticle surface environment, resulting in a reduction in ZP to approximately −24 mV for all samples, compared to their ZP in deionized water (day 0); however, no significant differences were observed between 2 and 24 h incubation timepoints in cell culture media. In terms of particle size distribution, most samples showed no significant differences between 2 and 24 hour incubation timepoints, with sizes remaining within the range reported in deionized water, while the PDI increased above 0.2 (Fig. S2†). Together, these findings support the need for further comprehensive exploration of biocompatibility and stress conditions to assess silk nanoparticle stability and drug delivery potential.

Given that silk nanoparticles in deionized water and sodium phosphate buffer exhibited no significant signs of agglomeration during short-term stability studies (2 hours), the long-term storage stability of nanoparticles in these media was investigated for up to 56 days at 4 °C. Silk nanoparticles dispersed in deionized water had excellent stability, with no changes in physicochemical properties observed (*i.e.*, particle size, concentration, PDI, and charge) relative to day 0. In contrast, a small change in these parameters was observed in sodium phosphate buffer, with increased particle size, reduced particle concentration, and changes in PDI. However, no changes in zeta potential were detected under these conditions.

Loss of colloidal stability was observed from day 7, particularly in the case of smaller-sized silk nanoparticles (82.7–97.3 d.nm). One possible explanation for this observation is the occurrence of ionic strength-induced particle aggregation achieved by reducing electrostatic repulsion between particles and promoting cluster formation with a broader size distribution (Fig. 3a and 4a). These results align with the FE-SEM micrographs captured on day 56, which illustrated the presence of greater numbers of aggregated silk nanoparticles in comparison to day 0. Conversely, no significant changes were detected for nanoparticles stored in deionized water (Fig. 3b and 4b). We speculate that, in this case, the particle size (∼100 d.nm) and concentration (≥2 × 10^11^ particles/mL) may play a vital role in regulating the silk nanoparticle colloidal stability^39,49^ in sodium phosphate buffer at physiological pH, rather than the particle surface net charge. In the absence of an added counterion (Ca^2+^/K^+^), and at 37 °C, the 0 mg cation silk nanoparticles exhibited reduced physicochemical stability at day 7, appearing as micron-sized agglomerates (∼5 µm) with higher polydispersity and a lower net charge compared to the smaller-sized nanoparticles formulated at lower Ca^2+^ (0.7 mg) and K^+^ (1.1 mg) concentrations (Fig. S1†). These results indicate that Ca^2+^ and K^+^ may promote nanoparticle stability at higher storage temperatures by sustaining a repulsive hydration force on the particle surface,^50^ stabilizing the silk nanoparticle core structure *via* β-sheet content,^14^ and preventing particle agglomeration. Our thioflavin T assay confirmed improved hydrophobic core strength in the presence of counterions, as thioflavin T can recognise β-sheet structures.^51^ Ca^2+^ and K^+^ significantly increase thioflavin T entrapment in particles, while anti-solvent precipitation occurs (Fig. S3†).

Taken together, our findings indicate that Ca^2+^ showed greater promise than K^+^ for enhancing silk nanoparticle physical properties and gave rise to nanoparticles with higher stability under acidic conditions and elevated temperature, that exhibited a lesser extent of alteration in agglomeration behavior (DLS, NTA, and FE-SEM) and net surface charge (ELS). One possible explanation is that counterions modulate silk conformation during nanoparticle formation through the formation of: (1) transient salt bridges with negatively charged residues located at the hydrophilic spacer and N-terminus of silk H-chain,^23^ which could be presented on the nanoparticle surface, and (2) coordination complexes with other amino acid residues in hydrophobic region such as histidine,^23^ serine,^52^ and tyrosine,^53,54^ addressed as an organizing and stabilizing β-sheet formation (reviewed in^55^), which could be presented inside the nanoparticle core. Due to the differences in ionic radii between Ca^2+^ (0.99 Å) and K^+^ (1.33 Å),^56^ Ca^2+^ may exhibit a stronger bonding with carbonyl oxygen, thereby constructing a more appropriate network of π–π interacting tyrosines^53,54^ and a stronger interaction with negatively-charged residues.^57^ Therefore, we propose that these factors may give the Ca^2+^ nanoparticles a higher particle physical strength. An increased temperature drives protein denaturation/unfolding by disrupting hydrogen bonds, electrostatic interactions, and hydrophobic forces,^58,59^ as well as a denaturation caused by acidic pH (pH 5.8). This may initiate the leakage of cations from less intact K^+^ silk nanoparticles, particularly in the case of larger particles (17.3 mg K^+^ nanoparticles), resulting in reduced zeta potential and increased agglomeration (Fig. 2b, Table 1 and Fig. S1†).

Although we observed that counterion inclusion in silk nanoparticle formulations minimized agglomeration behavior for up to 56 days under refrigerated storage conditions, the resulting increase in particle size remained within acceptable specifications for nanomedicine-based applications (Fig. 3 and 4). Nanoparticles in the 100–500 nm size range contribute to passive accumulation in tumors *via* the enhanced permeability and retention (EPR) effect,^60^ while particles 600 nm in size can provide penetration ability to hair follicles, which is beneficial for dermal drug delivery.^61^ Therefore size tuning offers the potential to unlock a range of therapeutic applications beyond cancer.

### Analysis of silk nanoparticle physical stability at different timepoints using asymmetric flow field-flow fractionation with online UV-multiangle light scattering and dynamic light scattering (AF4-UV-MALS-DLS)

Asymmetric flow field-flow fractionation (AF4) was used to investigate the particle size distribution profile, interparticle interactions, and the shape factor of silk nanoparticles during storage.

When stored in deionized water, silk nanoparticles formulated with 0.7 mg Ca^2+^ and 1.1 mg K^+^ exhibited a shift in their main elution peak (P1) relative to the 0 mg nanoparticles, from 18.1 min to 21.0 and 19.1 min, respectively. This indicated a larger size, with an increase in *R*_g_ from 25.5 nm to 26.1 nm and 25.6 nm, respectively. Interestingly, the 0.7 mg Ca^2+^ nanoparticles exhibited a higher MALS signal at ∼33 min compared to the other silk nanoparticles, indicating the presence of a larger number of larger particles and/or aggregates at peak 2 (P2) with *R*_g_ ∼ 28.0 nm. However, control silk nanoparticles (0 mg cation) and 1.1 mg K^+^ silk nanoparticles with smaller *R*_g_ ∼ 26.1 nm. After dispersal for 56 days in deionized water, the RT of P1 was slightly shorter than at day 0 (*R*_g_ in parentheses), eluting at 17.7 min, 19.6 min, and 18.3 min for 0 mg cation nanoparticles (24.7 nm), 0.7 mg Ca^2+^ silk nanoparticles (24.8 nm), and 1.1 mg K^+^ nanoparticles (24.9 nm), respectively. A noticeable shift in the RT of P2 was observed in the 0 mg cation silk nanoparticles (27.8 nm), increasing from 32.7 min to 38.4 min, whereas no significant changes in RT for P2 were observed in the other samples (∼33 min). Interestingly, the *R*_g_ values for 0.7 mg Ca^2+^ silk nanoparticles and 1.1 mg K^+^ silk nanoparticles were smaller at 25.6 nm and 25.0 nm, respectively (Fig. 3c and Table 2). After storage for 56 days, slight changes in the percentage recovery were evident in the 0 mg cation nanoparticles (85.79% to 82.99%) and 0.7 mg Ca^2+^ nanoparticles (92.94% to 75.30%). In contrast, the 1.1 mg K^+^ nanoparticles exhibited an optimal percent recovery, at 92.61% to 95.51%, until 56 days of storage (Fig. S4†).

**Table 2:** Parameters measured for silk nanoparticles dispersed in deionized water derived from electrical asymmetric flow field-flow fractionation (EAF4)-MALS. Two-way ANOVA and Šídák's multiple comparisons test were used in statistical analysis, comparing day 56 and 0 data; *p* < 0.05 (*), *p* < 0.01 (**), *p* < 0.001 (***), and *p* < 0.0001 (****). Abbreviations: peak 1 (P1); peak 2 (P2); hydrodynamic radius (*R*_h_); radius of gyration (*R*_g_); dynamic light scattering (DLS); zeta potential (ZP)

Silk nanoparticle	Applied current (mA)	Elution time (min)		MALS-*R*_g_ (nm)		DLS-*R*_h_ (nm)		*R*_g_/*R*_h_		Electrophoretic mobility (mm cm V^−1^ s^−1^)	Correlation coefficient (*R*^2^)	ZP (mV)
		P1	P2	P1	P2	P1	P2	P1	P2			
**Day 0**												
0 mg cation	+0.2	17.3 (±0.0)	32.8 (±0.5)	25.5 (±0.1)	25.9 (±0.0)	38.2 (±1.6)	53.8 (±15.8)	0.67 (±0.03)	0.51 (±0.14)	−4.71	0.9841	−60.30
	0	18.1 (±0.1)	32.7 (±0.2)	25.5 (±0.1)	26.1 (±0.0)	36.7 (±1.8)	67.1 (±19.9)	0.70 (±0.04)	0.42 (±0.41)			
	−0.2	18.9 (±0.1)	32.5 (±0.1)	25.6 (±0.1)	26.2 (±0.1)	39.7 (±5.3)	62.9 (±1.9)	0.65 (±0.09)	0.42 (±0.01)			
0.7 mg Ca^2+^	+0.2	20.1 (±0.4)	32.7 (±0.2)	26.2 (±0.1)	27.5 (±0.2)	44.2 (±2.0)	85.0 (±5.8)	0.59 (±0.03)	0.33 (±0.03)	−4.78	0.9864	−61.24
	0	21.0 (±0.1)	33.4 (±0.4)	26.1 (±0.0)	28.0 (±0.2)	46.5 (±1.0)	78.1 (±10.6)	0.56 (±0.01)	0.36 (±0.05)			
	−0.2	22.0 (±0.2)	33.3 (±0.1)	26.0 (±0.1)	28.0 (±0.1)	46.4 (±3.4)	71.4 (±9.8)	0.56 (±0.04)	0.40 (±0.06)			
1.1 mg K^+^	+0.2	18.0 (±0.1)	32.5 (±0.1)	25.5 (±0.1)	25.9 (±0.1)	42.5 (±1.7)	85.8 (±7.2)	0.60 (±0.02)	0.30 (±0.03)	−4.78	0.9795	−61.15
	0	19.1 (±0.1)	32.7 (±0.3)	25.6 (±0.1)	26.1 (±0.1)	43.3 (±1.0)	63.9 (±6.9)	0.70 (±0.01)	0.41 (±0.05)			
	−0.2	20.1 (±0.1)	32.8 (±0.1)	25.6 (±0.1)	26.4 (±0.1)	42.6 (±4.7)	64.6 (±15.3)	0.61 (±0.07)	0.42 (±0.10)			

**Day 56**												
0 mg cation	+0.2	16.8 (±0.2)*	37.7 (±0.1)****	24.0 (±1.2)****	28.1 (±0.2)*	42.2 (±0.9)	5.5 (±0.7)****	0.57 (±0.04)*	5.12 (±0.56)****	−4.84	0.9734	−62.02
	0	17.7 (±0.1)	38.4 (±0.1)****	24.7 (±0.1)*	27.8 (±0.1)	44.8 (±0.5)**	37.9 (±21.3)*	0.55 (±0.01)***	0.88 (±0.40)			
	−0.2	18.6 (±0.2)	39.0 (±0.1)****	24.7 (±0.1)**	23.6 (±5.0)**	43.1 (±0.7)	101.4 (±23.1)***	0.57 (±0.01)	0.24 (±0.11)			
0.7 mg Ca^2+^	+0.2	18.0 (±0.2)****	33.7 (±0.2)****	24.8 (±0.1)****	25.6 (±0.1)	49.0 (±2.2)	25.9 (±6.4)****	0.51 (±0.02)	1.03 (±0.29)	−4.22	0.9873	−54.07
	0	19.6 (±0.1)****	33.3 (±0.1)	24.8 (±0.1)****	25.6 (±0.1)*	49.0 (±2.0)	29.2 (±2.7)****	0.51 (±0.02)	0.88 (±0.08)			
	−0.2	20.7(±0.2)****	33.7 (±0.2)	24.9 (±0.0)****	25.9 (±0.1)*	49.4 (±1.7)	40.4 (±5.8)**	0.50 (±0.02)	0.66 (±0.09)			
1.1 mg K^+^	+0.2	17.6 (±0.2)*	32.8 (±0.2)	24.8 (±0.1)*	25.1 (±0.1)	44.2 (±1.4)	4.0 (±2.3)****	0.56 (±0.02)	5.52 (±4.29)****	−3.74	0.8712	−47.95
	0	18.3 (±0.4)****	32.9 (±0.2)	24.9 (±0.1)*	25.0 (±0.1)	44.9 (±1.0)	8.4 (±1.1)****	0.55 (±0.01)	3.02 (±0.40)*			
	−0.2	19.3 (±0.1)****	33.3 (±0.1)*	24.8 (±0.0)**	25.2 (±0.0)	45.3 (±1.1)	13.4 (±6.3)****	0.59 (±0.01)	2.73 (±1.33)			

AF4 analysis provided a more comprehensive insight into interparticle interactions and particle structure, indicating a minor yield of aggregates during analysis in 0.5 mM Na_2_CO_3_ (∼pH 10) for the 0.7 mg Ca^2+^ silk nanoparticles rather than for the control (0 mg cation) and 1.1 mg K^+^ silk nanoparticles. The same result was not detectable using DLS or NTA measurements; however, it was consistent with a colloidal stability test under basic pH conditions, with dispersal in sodium phosphate buffer (pH 8.0), where at elevated pH, a high degree of aggregation (more negative *k*_D_) of the 0.7 mg Ca^2+^ nanoparticles was observed, whereas the other silk nanoparticles showed an opposite trend (less negative *k*_D_) (Table 1). DLS measurement of the particle size for the silk nanoparticles dispersed in 0.5 mM Na_2_CO_3_ (∼pH 10) for 1 h, which mimics the duration of the AF4 analysis, showed particle growth compared to those dispersed in sodium phosphate buffer (pH 8.0), with an increase in Z-average from 121.4 d.nm to 142.8 d.nm for the 0.7 mg Ca^2+^ nanoparticles. In contrast, a negligible increase in Z-average was observed for the 0 mg cation nanoparticles (from 112.3 d.nm to 116.2 d.nm) and the 1.1 mg K^+^ nanoparticle (from 113.3 d.nm to 126.7 d.nm) (Fig. S5a†). We speculate that a high pH (pH 8–10) reduced the stability of the 0.7 mg Ca^2+^ nanoparticles to a greater extent than for the other silk nanoparticles through several possible pathways, such as protein denaturation, particle swelling, and net charge surface alteration.^32–34^

The AF4-UV-MALS-DLS provided us with more insights into particle size and morphological properties. As the particle diffuses/moves through the solution, the radius of gyration (*R*_g_), measured by the inline MALS detector, along with a hydrodynamic radius (*R*_h_) measured by the inline DLS, can be determined. The hydrodynamic radius (*R*_h_) refers to the radius of a hard sphere that diffuses in the same way as the particle, so it includes the actual particle and the solvation shell. Therefore, a larger particle will experience a larger friction moving in solution and thus have a larger *R*_h_.^62,63^ The radius of gyration (*R*_g_) refers to the distribution of mass around the center of the particle mass, indicating a particle structure, as a compact particle will have a small *R*_g_ and an extended/loosened particle will have a larger *R*_g_.^64^ The ratio of *R*_g_ to *R*_h_, referred to as the shape factor, is commonly used to investigate the shape and retention-related properties of particles in solution. A spherical shape possesses an *R*_g_/*R*_h_ of approximately 0.755.^62^

**Fig. 5:**
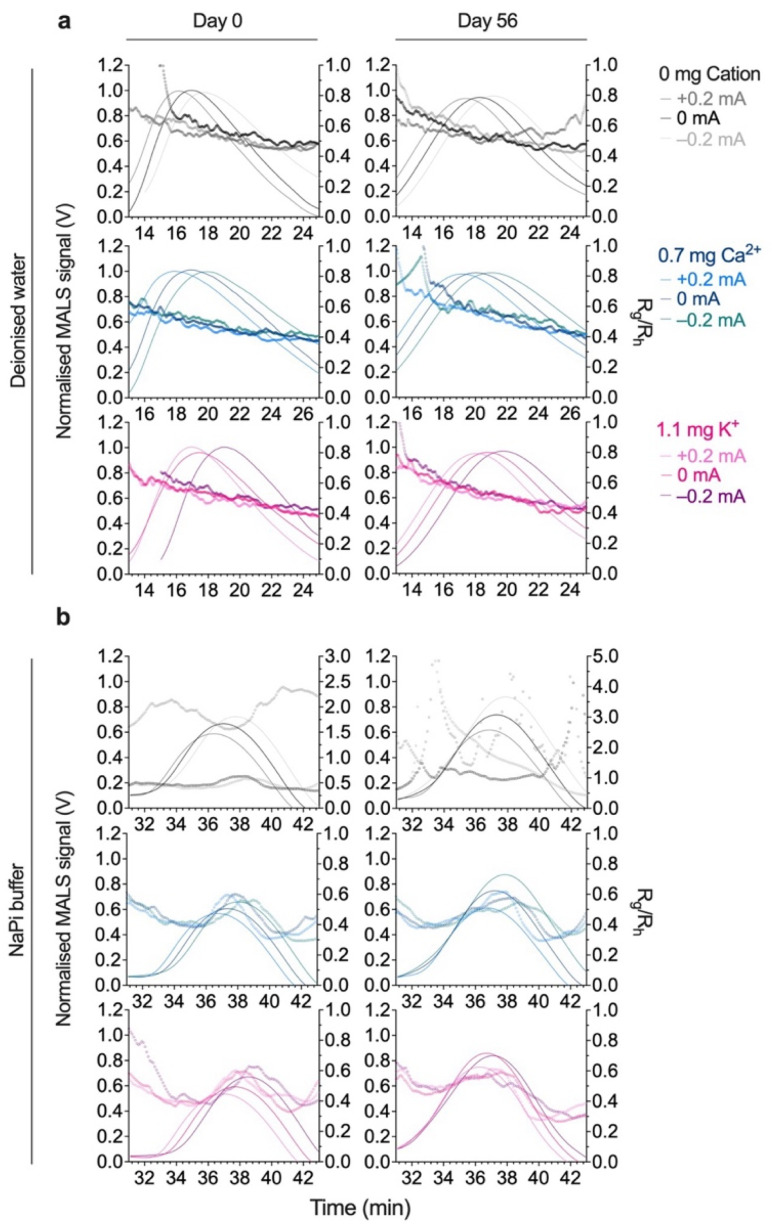
Electrical asymmetric flow field-flow fractionation (EAF4) fractograms of silk nanoparticles dispersed in deionized water and 10 mM sodium phosphate (NaPi) buffer (pH 7.4). Silk nanoparticles (0.1 mg/mL) were separated in 0.5 mM sodium carbonate under electrical fields with applied positive (+0.2 mA), neutral (0 mA), and negative (−0.2 mA) currents. (a) Nanoparticles EAF4 fractograms for day 0 and day 56 storage were presented as a function of normalized MALS signal and shape factor (*R*_g_/*R*_h_): (a) deionized water storage and (b) NaPi buffer (pH 7.4) storage (*n* = 3). Abbreviations: hydrodynamic radius (*R*_h_); radius of gyration (*R*_g_); multiangle light scattering (MALS).

The AF4 investigation study initially revealed a considerable impact of the counterions (Ca^2+^ and K^+^) on the retention, size, and shape of silk nanoparticles during separation in a flowing system. According to the separation profiles of the control (0 mg cation) and 1.1 mg K^+^ silk nanoparticles, particles with a more spherical shape and a tightly packed structure could potentially be separated from the crude silk particle population. These particles eluted as the major peak (P1), confirming their small *R*_g_ and *R*_h_ values, with shape factors close to the ideal value of 0.755.^62^ This indicated a high colloidal stability of nanoparticles even when exposed to high pH conditions for 1 h. However, the 0.7 mg Ca^2+^ nanoparticles exhibited noticeable colloidal instability (increased aggregation), together with a shape distortion that deviated from the ideal spherical shape (from 0.755 (ref. 55) to 0.56), and represented most particles eluting as P1. The evidence from this study suggests that Ca^2+^ incorporation into silk nanoparticles improved the structural stability and particle–solvent interaction to a greater extent than was possible with the control (0 mg cation) or 1.1 mg K^+^ silk nanoparticles at basic pH (∼pH 10). Loosening the particle structure (larger *R*_g_) and increasing the solvation layer (larger *R*_h_) led to a longer retention time (*i.e.*, longer traveling time in solution due to low diffusivity and more friction) (Fig. 3c, 5a, and Table 2).

Comparison of the valency state and ionic radii between Ca^2+^ and K^+^ reveals that Ca^+^ is more capable than K^+^ of forming strong charge–charge or electrostatic interactions with proteins.^53,54,56,57^ However, the charged residues on proteins under AF4 separation and the changes incurred by an increase in pH (silk becomes more negative)^41^ may corrupt the ordered silk conformation and/or silk–counterion interactions, thus reducing the particle integrity, distorting the particle shape, and promoting particle aggregation. However, after 56 days of storage, the 0.7 mg Ca^2+^ silk nanoparticles showed fewer aggregates compared to day 0. The alterations may have only a minor effect, leading to reversible, transient aggregates at day 56.

Interestingly, the shape-retention stability of the silk nanoparticles dispersed in deionized water, as demonstrated by the FE-SEM micrographs, showed fewer aggregates and smooth, round particles up to day 56 (Fig. 3b). Therefore, the inconsistency between the computed shape factor and the FE-SEM micrograph, especially for the 0.7 mg Ca^2+^ nanoparticles, may be due to an influence of the carrier liquid composition and pH during AF4 separation.

Silk nanoparticles dispersed in sodium phosphate buffer (pH 7.4) demonstrated different fractograms for the 0 mg cation, 0.7 mg Ca^2+^, and 1.1 mg K^+^ silk nanoparticles, which showed a broad peak of small *R*_g_ particles sized 24.5 nm at 18.9 min (24.5 nm), 24.6 nm at 22.1 min, and 24.4 nm at 21.5 min, respectively, and a sharp peak of large *R*_g_ particles sized 45.5 nm at 25.9 min, 51.9 nm at 26.6 min, and 55.5 nm at 29.0 min (55.5 nm), respectively. After 56 days, the 0.7 mg Ca^2+^ silk nanoparticles showed significant changes in the RT of P1, which shifted to 28.0 nm at 26.6 min, whereas P2 showed no significant changes, eluting at 38.0 min with a size of 59.1 nm. However, the RTs for P1 and P2 of the other silk nanoparticles were shifted to 30.3 min (P1 particle size: 29.5 nm) and 38.1 min (P2 size: 61.8 nm) for 0 mg cation and to 29.0 min (P1 size: 29.0 nm) and 37.8 min (P2 size: 50.0 nm) for 1.1 mg K^+^. Importantly, the *R*_g_ of the silk nanoparticles showed no significant difference after 56 days (Fig. 4c and Table 3). Similar to particles dispersed in water, the 0.7 mg Ca^2+^ and 1.1 mg K^+^ silk nanoparticles dispersed in sodium phosphate buffer (pH 7.4) exhibited >82% recoveries from day 0 until day 56, while the 0 mg cation silk nanoparticles had percent recoveries of ∼70% (Fig. S4†).

**Table 3:** Parameters measured for silk nanoparticles dispersed in 10 mM sodium phosphate buffer (pH 7.4) derived from electrical asymmetric flow field-flow fractionation (EAF4)-MALS. Two-way ANOVA and Šídák's multiple comparisons test were used, comparing day 56 and 0 data: *p* < 0.05 (*), *p* < 0.01 (**), *p* < 0.001 (***), and *p* < 0.0001 (****). Abbreviations: peak 1 (P1); peak 2 (P2); hydrodynamic radius (*R*_h_); radius of gyration (*R*_g_); dynamic light scattering (DLS); zeta potential (ZP); no data available (NA)

Silk nanoparticle	Applied current (mA)	Elution time (min)		MALS-*R*_g_ (nm)		DLS-*R*_h_ (nm)		*R*_g_/*R*_h_	
		P1	P2	P1	P2	P1	P2	P1	P2
**Day 0**									
0 mg cation	+0.2	17.7 (±0.4)	37.0 (±0.1)	24.6 (±0.1)	46.1 (±0.1)	33.7 (±2.6)	73.4 (±13.9)	0.73 (±0.06)	0.64 (±0.14)
	0	18.9 (±0.3)	37.7 (±0.1)	24.5 (±0.1)	45.5 (±1.0)	35.9 (±9.8)	79.1 (±3.3)	0.73 (±0.23)	0.57 (±0.01)
	−0.2	20.2 (±0.9)	38.4 (±0.1)	24.4 (±0.1)	45.8 (±0.6)	31.6 (±4.3)	76.4 (±16.4)	0.78 (±0.10)	0.62 (±0.14)
0.7 mg Ca^2+^	+0.2	21.3 (±0.2)	37.3 (±0.1)	24.7 (±0.1)	51.7 (±0.3)	38.0 (±5.3)	94.5 (±5.3)	0.66 (±0.10)	0.64 (±0.11)
	0	22.1 (±0.1)	37.9 (±0.1)	24.6 (±0.1)	51.9 (±0.3)	33.5 (±9.1)	81.8 (±12.8)	0.77 (±0.18)	0.55 (±0.03)
	−0.2	23.3(±0.3)	38.5 (±0.0)	24.6 (±0.1)	50.0 (±0.3)	31.6 (±3.7)	87.5 (±4.5)	0.79 (±0.09)	0.57 (±0.03)
1.1 mg K^+^	+0.2	20.4 (±0.4)	37.5 (±0.0)	24.7 (±0.1)	57.7 (±0.6)	50.4 (±9.2)	89.2 (±6.3)	0.50 (±0.10)	0.65 (±0.04)
	0	21.5 (±0.5)	38.2 (±0.0)	24.4 (±0.1)	55.5 (±0.4)	31.6 (±4.1)	93.0 (±0.7)	0.78 (±0.10)	0.60 (±0.01)
	−0.2	22.7 (±0.3)	38.8 (±0.1)	24.3 (±0.1)	53.2 (±0.6)	25.3 (±2.4)	85.3 (±5.1)	0.97 (±0.10)	0.63 (±0.03)

**Day 56**									
0 mg cation	+0.2	25.9 (±0.1)****	37.5 (±0.1)****	27.9 (±0.3)****	62.2 (±1.3)****	31.4 (±14.0)	8.0 (±5.8)****	0.99 (±0.35)	18.30 (±21.95)**
	0	30.3 (±0.3)****	38.1 (±0.1)****	29.5 (±0.3)****	61.8 (±0.3)****	56.5 (±11.7)	78.3 (±42.6)	0.54 (±0.12)	0.94 (±0.44)
	−0.2	29.8 (±0.8)****	38.7 (±0.1)****	29.3 (±0.3)****	56.7 (±0.3)****	65.9 (±34.4)*	136.7 (±108.8)****	0.57 (±0.37)	1.38 (±1.88)
0.7 mg Ca^2+^	+0.2	26.3 (±0.5)****	37.5 (±0.1)*	27.9 (±0.3)****	62.5 (±1.4)****	45.0 (±6.1)	102.7 (±2.3)	0.63 (±0.08)	0.61 (±0.02)
	0	26.6 (±0.0)****	38.0 (±0.1)	28.0 (±0.2)****	59.1 (±0.6)****	48.3 (±19.9)	103.5 (±10.2)	0.64 (±0.21)	0.57 (±0.05)
	−0.2	26.6 (±0.0)****	38.7 (±0.0)**	27.8 (±0.1)****	54.3 (±0.4)****	39.7 (±9.9)	99.9 (±5.7)	0.73 (±0.17)	0.54 (±0.03)
1.1 mg K^+^	+0.2	28.4 (±0.3)****	37.3 (±0.1)*	29.5 (±0.2)****	52.2 (±0.5)****	42.2 (±4.1)	102.7 (±2.3)	0.70 (±0.07)	0.51 (±0.01)
	0	29.0 (±0.4)****	37.8 (±0.0)****	29.1 (±0.1)****	50.0 (±0.3)****	52.9 (±19.1)	103.7 (±9.0)	0.61 (±0.25)	0.48 (±0.04)
	−0.2	29.6 (±0.6)****	38.2 (±0.1)****	29.0 (±0.2)****	46.1 (±0.2)	45.5 (±7.4)	102.1 (±5.1)	0.65 (±0.11)	0.45 (±0.02)

The silk nanoparticle fractograms studied with the UV 280 nm signal showed a correlation between the UV signal and the particle size data for both *R*_g_ and *R*_h_ (Fig. S6, Tables S2 and S3†). However, the particle size for *R*_g_ (*R*_h_ in parentheses) in the range of 25.5–26.1 nm (36.7–43.4 nm) accounted for the majority of nanoparticles dispersed in deionized water, indicating a narrow size distribution of uniform and non-aggregated particles. In contrast, an increased particle size was observed in sodium phosphate buffer (pH 7.4), with both *R*_g_ and *R*_h_ increasing ∼2-fold for each sample. We hypothesized that the physicochemical attributes of the silk nanoparticles dispersed in sodium phosphate buffer (pH 7.4) had undergone a slight change based on the alteration in diffusivity shown in Fig. 2a (increased *R*_h_), as well as a loss in particle integrity (increased *R*_g_) and an increase in particle agglomeration, as shown in Fig. 4b (increased *R*_g_ and *R*_h_). Interestingly, the shape factor was still maintained in deionized water (ranging from 0.55–0.65), suggesting a slight deviation from a spherical shape (Fig. 5b and Table 3). Storage for 56 days significantly altered the physicochemical stability of the 0 mg cation nanoparticles to a greater extent than was observed for the 0.7 mg Ca^2+^ and 1.1 mg K^+^ silk nanoparticles stored in sodium phosphate buffer (pH 7.4) buffer. The 0 mg cation nanoparticles changed significantly in size (*R*_g_ and *R*_h_) and shape (>1) (Fig. 5b and Table 3). A potential reason for this was the lower colloidal stability of the 0 mg cation nanoparticles (with a lower *k*_D_ of −2.373) (Table 1). In the absence of counterions (Ca^2+^ and K^+^), the repulsive interactions between particles and the particle–solvent interactions were weakened and/or unsteady, leading to enhanced agglomeration, fluctuating *R*_g_ and *R*_h_ values, and an extended shape. Conversely, the silk nanoparticles with 0.7 mg Ca^2+^ and 1.1 mg K^+^, when stored in sodium phosphate buffer (pH 8.0), exhibited excellent physical stability in terms of both size and shape retention, as reflected in their flow behaviors, packing densities, and surface areas. Almost certainly, our findings will help in predicting silk nanoparticle behavior in different environments, in designing and developing nanoparticles with controlled and favorable physicochemical attributes, and in optimizing nanoparticles for various industrial purposes.

### Evaluation of silk nanoparticle surface charges using electrical asymmetric flow field-flow fractionation (EAF4-UV-MALS-DLS)

The EAF4 fractograms presented include an overlay of a normalized MALS signal together with the shape factor (*R*_g_/*R*_h_). Silk nanoparticles were separated based on their size (diffusion) and charge (zeta potential) properties under an electric field created by applying positive (+0.2 mA) and negative (−0.2 mA) currents. Silk nanoparticles exhibited a slight shift of the first peak (P1) to a lower elution time when applying a positive current and a shift in the opposite direction toward a higher RT when applying a negative current.

In deionized water, a shift of P1 that deviated from the neutral current condition was evident for all silk nanoparticles: 0 mg cation nanoparticles: 18.1 min to 17.3 min (+0.2 mA) and 18.9 min (−0.2 mA); 0.7 mg Ca^2+^ nanoparticles: 21.0 min to 20.1 min (+0.2 mA) and 22.0 min (−0.2 mA); and 1.1 mg K^+^ nanoparticles: 19.1 min to 18.0 min (+0.2 mA) and 20.1 min (−0.2 mA). However, only negligible changes were seen in the RT in response to the electrical field for P2 (32.5–33.3 min) (Fig. 5a and Table 2). Furthermore, no significant differences were detected in the computed particle size of P1 (*R*_g_ = 25.5–26.2 nm and *R*_h_ = 36.7–46.5 nm) under different applied currents. The 0.7 mg Ca^2+^ silk nanoparticles showed noticeable similar properties to those observed in the absence of an electric field, with agglomerates appearing after an elution time of ∼33 min (P2), an *R*_g_ range of 27.5–28.0 nm, and an *R*_h_ range of 71.4–85.0 nm, while the 0 mg cation and 1.1 mg K^+^ nanoparticles were smaller in size, with corresponding *R*_g_ ranges of 25.9–26.4 nm and *R*_h_ ranges of 53.8–85.8 nm (Fig. S4a and b:† upper panel and Table 2).

Nanoparticle shape factor was determined using the *R*_g_/*R*_h_ ratio, with 0 mg cation and 1.1 mg K^+^ nanoparticles having shape factors of 0.60–0.70, while 0.7 mg Ca^2+^ nanoparticles had a lower shape factor value of 0.56–0.59. These data indicate that nanoparticles had a predominantly spherical morphology, as a homogenous hard sphere has a shape factor ∼0.755.^62^ Larger aggregates exhibited a lower shape factor value, consistent with the FE-SEM results (Fig. 3b). Interestingly, a greater deviation from 0.755 was detected for P2, ranging from 0.30–0.51 on average (Fig. 5a and Table 2).

The zeta potential of silk nanoparticles was estimated using EAF4-UV-MALS. The 0 mg cation silk nanoparticles exhibited the lowest zeta potential at −60.30 mV, and no considerable differences were observed between 0.7 mg Ca^2+^ and 1.1 mg K^+^ nanoparticles, which showed zeta potentials at −61.24 mV and −61.15 mV, respectively (Table 2). After 56 days, the zeta potential decreased to −62.02 mV, −54.07 mV, and −47.95 mV for 0 mg Cation, 0.7 mg Ca^2+^, and 1.1 mg K^+^ nanoparticles, respectively. Interestingly, the ELS data indicated less negative zeta potentials for silk nanoparticles dispersed in 0.5 mM Na_2_CO_3_ (∼pH 10) for 1 h, at −37.03 mV for 0 mg cation nanoparticles, −34.33 mV for 0.7 mg Ca^2+^ nanoparticles, and −33.07 mV for 1.1 mg K^+^ nanoparticles (Fig. S5b†). The percentage recovery of the measured fractions was calculated relative to the direct injection method, with recovery rates of 86 (±7)%, 93 (±3)%, and 93 (±8)% achieved for the 0 mg cation, 0.7 mg Ca^2+^, and 1.1 mg K^+^ nanoparticles, respectively.

After 56 days of storage, the silk nanoparticles dispersed in deionized water maintained desirable responsiveness under different applied currents, displaying similar shifting profiles in RT for P1; 0 mg cation nanoparticles: 17.7 min to 16.8 min (+0.2 mA) and 18.6 min (−0.2 mA), 0.7 mg Ca^2+^ nanoparticles: 19.6 min to 18.0 min (+0.2 mA) and 20.7 min (−0.2 mA), and 1.1 mg K^+^ nanoparticles: 18.3 min to 17.6 min (+0.2 mA) and 19.3 min (−0.2 mA) and with a negligible shift for P2 (0 mg cation:37.7–39.0 min, 0.7 mg Ca^2+^ and 1.1 mg K^+^ silk nanoparticles: 32.8–33.7 min). However, most RTs reported on day 56 were significantly different from those on day 0 (Fig. S4a and b:† upper panel and Table 2). The prominent reduction in *R*_g_ was mainly observed in P1 rather than P2, whereas the *R*_h_ (in parentheses) fluctuated between +0.2 mA and −0.2 mA; 0 mg cation silk nanoparticles (53.8–62.9 nm to 5.5–101.4 nm), 0.7 mg Ca^2+^ nanoparticles (71.4–85.0 nm to 25.9–40.4 nm) and 1.1 mg K^+^ nanoparticles (63.9–85.8 nm to 4.0–13.4 nm). These changes in *R*_g_ and *R*_h_ altered the shape factor profile, increasing the shape factor in P2 significantly: 0 mg cation silk nanoparticles (from 0.45–0.51 to 0.24–5.12), 0.7 mg Ca^2+^ nanoparticles (from 0.33–0.40 to 0.66–1.03), and 1.1 mg K^+^ nanoparticles (from 0.30–0.42 to 2.73–5.52). Interestingly, the positive current altered the particle morphology, increasing the shape factor to over ∼0.755, indicating a hollow sphere and/or a rod shape (Fig. 5a and Table 2).^62,63^ The recovery percentage of silk nanoparticles on day 56 changed only slightly from day 0, with recovery rates of 83 (±2)%, 75 (±11)%, and 96 (±7)% for 0 mg cation, 0.7 mg Ca^2+^, and 1.1 mg K^+^ nanoparticles, respectively.

In contrast to earlier findings, silk nanoparticles dispersed in NaPi buffer (pH 7.4) exhibited significant alterations in the fractograms, revealing a different majority of larger particles eluting as P2. Shifted apical peaks for P1 and P2 in response to the electrical field were also observed. The shifting in P1 for 0 mg cation nanoparticles was from 18.9 min to 17.7 min (+0.2 mA) and 20.2 min (−0.2 mA), for the 0.7 mg Ca^2+^ nanoparticles, it was from 22.1 min to 20.4 min (+0.2 mA) and 23.3 min (−0.2 mA), and for the 1.1 mg K^+^ nanoparticles it was from 21.5 min to 20.4 min (+0.2 mA) and 22.7 min (−0.2 mA). The shift in P2 was detected as a longer RT: for 0 mg cation nanoparticles, from 37.7 min to 37.0 min (+0.2 mA) and 38.4 min (−0.2 mA), for the 0.7 mg Ca^2+^ nanoparticles, from 37.9 min to 37.3 min (+0.2 mA) and 38.5 min (−0.2 mA), and for the 1.1 mg K^+^ nanoparticles, from 38.2 min to 37.5 min (+0.2 mA) and 38.8 min (−0.2 mA). On day 56, significant differences in RT were recorded, with a longer RT ranging from 25.9–0.3 min and 37.3–38.7 min for P1 and P2, respectively (Fig. 5b and Table 3).

The *R*_g_ and *R*_h_ for P1 identified under deionized water conditions showed *R*_g_ and *R*_h_ in the ranges of 27.8–29.5 nm and 31.4–65.9 nm, respectively. These values were significantly different from the values on day 0. The *R*_g_ and *R*_h_ (in parentheses) for P2 were higher than for the majority of particles dispersed in deionized water, with particle sizes of 45.5–46.1 nm (73.4–79.1 nm), 50.0–51.9 nm (81.8–94.5 nm), and 52.3–57.7 nm (85.3–93.0 nm) for 0 mg cation, 0.7 mg Ca^2+^, and 1.1 mg K^+^ nanoparticles, respectively. The *R*_g_ after 56 days was similar; however, no significant changes in *R*_h_ were detected for the 0.7 mg Ca^2+^ and 1.1 mg K^+^ nanoparticles after 56 days of storage, whereas the 0 mg cation nanoparticles showed a wide range of *R*_h_ values 8–136.7 nm (Fig. S4a and b:† lower panel and Table 3). The shape factor of the 0 mg cation nanoparticles was clearly influenced, as the nanoparticles displayed an increased and fluctuating shape factor value from day 0 (0.57–0.64) to day 56 (0.94–18.3). Retention of particle morphology for the 0.7 mg Ca^2+^ and 1.1 mg K^+^ nanoparticles was greater, with shape factors of ∼0.55–0.64 and 0.45–0.61 for day 0 and day 56, respectively. Smaller-sized particles were indicated, as P1 showed no significant changes in shape factor, ranging from 0.50 to 0.70, during storage (Fig. S4a and b:† lower panel and Table 3).

Assessment of the recovery percentage of nanoparticles dispersed in sodium phosphate buffer (pH 7.4) revealed recovery rates of 70 (±6)% for 0 mg cation, 82 (±6)% for 0.7 mg Ca^2+^, and 92 (±7)% for 1.1 mg K^+^ nanoparticles, however; the rate was slightly changed after 56 days, at 74 (±9)%, 87 (±3)%, and 87 (±5)%, respectively. The zeta potential could not be calculated here because the main peak was eluted in the absence of a cross flow. Silk nanoparticle fractograms detected with the UV 280 detector were conducted under ±0.2 mA currents and are shown along with the particle size data (*R*_g_ and *R*_h_) in the supplementary section (Fig. S4, Tables S1 and S2†).

EAF4, which combines AF4 and electrical field-flow fractionation, provides charge- and size-dependent separation of analytes.^19,65^ Therefore, using EAF4, nanoparticle characteristics can be further probed in response to the application of an electric field, with nanoparticles expected to exhibit significant responses to an applied electrophoretic force under a ±0.2 mA current to the top electrode, which would shift the main elution peak (P1) relative to neutral conditions (absence of applied current, 0 mA) (Fig. 5). Our findings suggest that the positive top electrode (+0.2 mA) accelerates the movement of silk nanoparticles toward the membrane according to the particle size and charge density while simultaneously increasing the negative surface charge of the membrane. This shortens the retention time due to increased electrostatic repulsion effects between silk nanoparticles and the membrane.^66^ Conversely, applying a negative current to the top electrode (−0.2 mA) alters the nanoparticle–membrane interaction and increases the electrostatic attractive forces generated by the bottom electrode, resulting in a longer retention time.^66^ Interestingly, electrophoretic force appeared to have the most significant impact on 0.7 mg Ca^2+^ nanoparticles, resulting in significant separation profiles by decreasing the proportion of nanoparticles under an applied positive current and promoting agglomeration under an applied negative current.

The zeta potential of the 0.7 mg Ca^2+^ nanoparticles was less negative than that of the control silk (0 mg cation) and 1.1 mg K^+^ nanoparticles. These properties support the assumption that an applied positive current of 0.2 mA may induce aggregation and adsorption of 0.7 mg Ca^2+^ nanoparticles relative to the neutral and applied negative current conditions. These data were aligned with the particle characteristics of 0.7 mg Ca^2+^ nanoparticles studied with AF4 and revealed the formation of a transient nanoparticle complex. However, we suggest that this represents a minor issue for silk nanoparticle formulation and characterization, as it resulted in a high recovery percentage of >80%.

Our EAF4 data confirmed the role of counterions (Ca^2+^ and K^+^) in stabilizing the colloidal stability of silk nanoparticles during storage in both deionized water and sodium phosphate buffer (pH 7.4). When the nanoparticles were observed under the electric field, the EAF4 analysis revealed the particle properties in response to charge and strengthened the AF4 analysis by showing that the counterions play a crucial role in improving particle interactions through their charge properties (resulting in fewer/transient aggregates). This also helps to retain particle structure and shape of the majority of the silk nanoparticles (shape factor: 0.50–0.59 for deionized water and 0.48–0.61 for sodium phosphate buffer at pH 7.4). In contrast, a noticeable distortion in particle shape and size was routinely observed for the control silk nanoparticles (0 mg cation) (Fig. 5, Tables 2 and 3).

Taken together, our EAF4 findings indicated favorable properties for silk nanoparticles separated by particle size and charge. We propose that these findings reveal the interparticle interactions occurring in a flowing system as well as the particle–membrane interaction, thereby reflecting the promising biochemical functions of negatively charged nanoparticles in clinical situations, such as intratumoral distribution enhancement (reviewed in^67^) and skin penetration enhancement (reviewed in^68^). Due to the limited range of carrier liquids that can be used for EAF4 applications, further investigations into carrier liquid composition and its impact on EAF4 analyses are required to develop more relevant mobile phases for charge-based separation of nanocarriers.

## Conclusions

We used orthogonal analytical methods to investigate the physical stability of silk nanoparticles formulated with cations (Ca^2+^ and K^+^). All nanoparticles exhibited acceptable short-term physical stability when dispersed in deionized water and phosphate buffer. Further profiling of the long-term storage stability of these nanoparticles revealed negligible changes in particle size and charge following storage in deionized water, but significant changes were observed by day 7 following storage in sodium phosphate buffer (pH 7.4). This study reports the first use of conventional and electric asymmetric flow field flow fraction modalities with inline light scattering to probe changes in nanoparticle size, charge, and shape. We presented water-based storage at 4 °C, 25 °C, and 37 °C to reflect relevant physiological and formulation conditions (Fig. S1†), enabling assessment of temperature-driven stability without ionic interference. Sodium phosphate buffer at 4 °C was included to evaluate aggregation under ionic stress at low temperature. Overall, our findings suggest that silk nanoparticles formulated with a low cation content of 0.7 mg Ca^2+^ or 1.1 mg K^+^ exhibit superior physicochemical stability, making them promising candidates for drug nanocarrier development. This design provides a foundational understanding of temperature and ionic effects on silk nanoparticle stability. Future work will investigate additional formulation and environmental stressors—including freeze–thaw, lyophilization, and agitation—, along with evaluations of drug-loaded silk nanoparticles at clinically relevant concentrations and with incorporated therapeutic agents to better assess their stability and suitability for drug delivery applications.

## Author contributions

NR contributed to drafting the manuscript, preparing samples, conducting experiments, and analyzing and graphing data. PP was responsible for training on the NTA and AF4 instruments, training associated with analysis software, and assisting in the method development for AF4. ZR and FPS were accountable for the study conceptualization and design, data analysis, manuscript revisions, and securing funding.

## Conflicts of interest

The authors declare no conflicts of interest.

## Supplementary Material

NA-007-D5NA00365B-s001

## Data Availability

All data created during this research are openly available from the University of Strathclyde-Pure, at https://doi.org/10.15129/31e72395-2d70-4896-aecd-5b2c0fb2897a.
