# Supplementary material for: Evaluating the impact of bioinspired counterion inclusion on silk nanoparticle physicochemical attributes and physical stability
Source: Nanoscale Adv. 2025 Jul 21;7(18):5519–35. doi: 10.1039/d5na00365b (PMC12302029; doi:10.1039/d5na00365b)
Supplement: NA-007-D5NA00365B-s001 [file NA-007-D5NA00365B-s001.pdf]

Supplementary Information (SI) for Nanoscale Advances.
This journal is © The Royal Society of Chemistry 2025

Evaluating the impact of bioinspired counterion inclusion on silk nanoparticle physicochemical attributes and physical stability

Napaporn Roamcharern[a], Panida Punnabhum[a], F. Philipp Seib[a,b,c], Zahra Rattray[a,*]

[a]Strathclyde Institute of Pharmacy and Biomedical Sciences, University of Strathclyde, 161 Cathedral St., Glasgow G4 0RE, Scotland, UK

[b]Fraunhofer Institute for Molecular Biology and Applied Ecology, Branch Bioresources, Ohlebergsweg 12, 35392 Giessen, Germany

[c]Friedrich Schiller University Jena, Institute of Pharmacy, Department of Pharmaceutical Technology and Biopharmaceutics, Lessingstr. 8, 07743 Jena, Germany

*Corresponding authors

Supplementary data

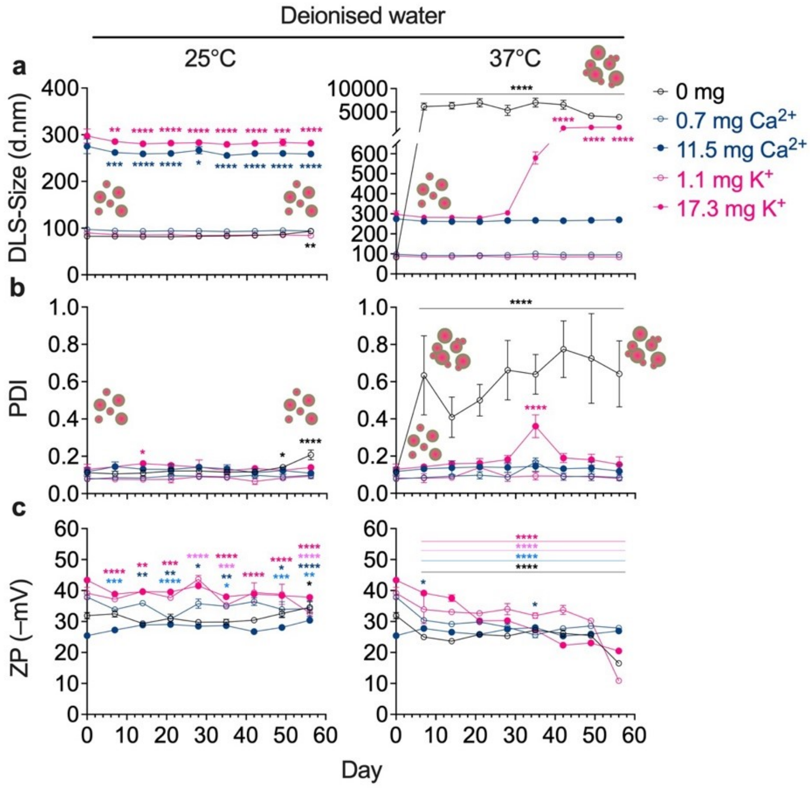

Fig. S1 Time storage stability of silk nanoparticles (SNPs) in water. (**a**) Particle size as measured by Dynamic Light Scattering: DLS, (**b**) particle concentration, (**c**) polydispersity index (PDI), and (**d**) zeta potential (ZP) measured over 56 storage days in deionized water at 25 °C and 37 °C. Two-way ANOVA and Dunnett's multiple comparisons test were assessed for statistical analyses comparable to day 0: $p < 0.05$ (*), $p < 0.01$ (**), $p < 0.001$ (***), and $p < 0.0001$ (****) (n = 3).

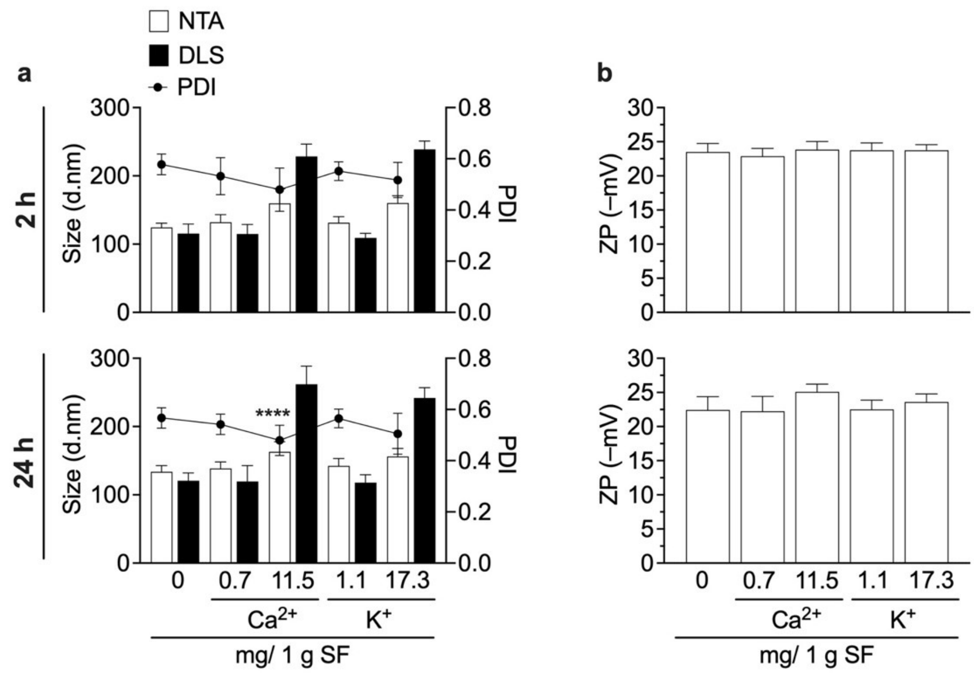

Fig. S2 Silk nanoparticle size and zeta potential in a biological-relevant system. Silk nanoparticles (1 mg/mL) were dispersed in complete media (Dulbecco's Modified Eagle Medium (DMEM) supplemented with 10% *v/v* FBS, 50 U/mL penicillin, and 50 mg/mL streptomycin), following by incubation at 37 °C for 2 and 24 h: (**a**) particle size *via* Dynamic light scattering (DLS) and Nanoparticle tracking analysis (NTA), and (**b**) zeta potential (ZP) *via* Electrophoretic light scattering (ELS). Two-way ANOVA and Šídák's multiple comparisons test were assessed for statistical analyses comparable to a 2-h incubation: $p < 0.05$ (*), $p < 0.01$ (**), $p < 0.001$ (***), and $p < 0.0001$ (****) ($n = 3$).

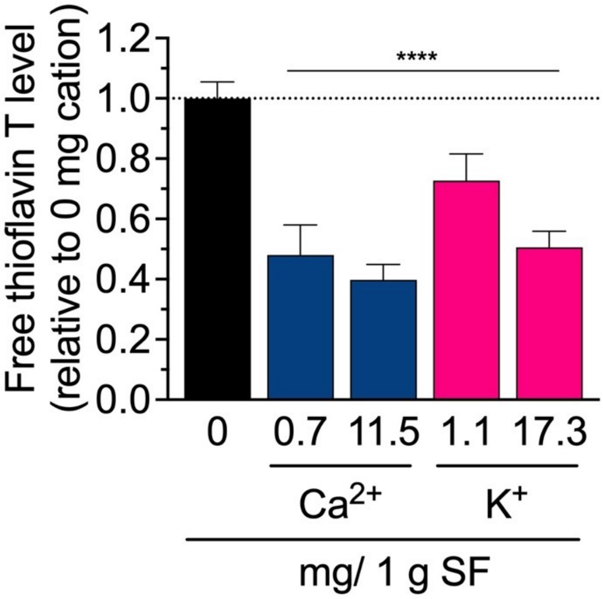

Fig. S3 Thioflavin T assay. Silk nanoparticle was fabricated by the anti-solvent precipitation method in a semi-batch with 3% *w/v* silk fibroin (SF) solution contained 100 μL of thioflavin T (1 mM). The reduction of free thioflavin T molecule was measured in a supernatant collected after centrifugation at 48,000*g* for 2 h, followed by a fluorescence reading at 460 nm (ex. 355 nm). One-way ANOVA and Dunnett's multiple comparisons test were assessed for statistical analyses comparable to a baseline 0 mg cation (dot line): $p < 0.05$ (*), $p < 0.01$ (**), $p < 0.001$ (***), and $p < 0.0001$ (****) (n = 3).

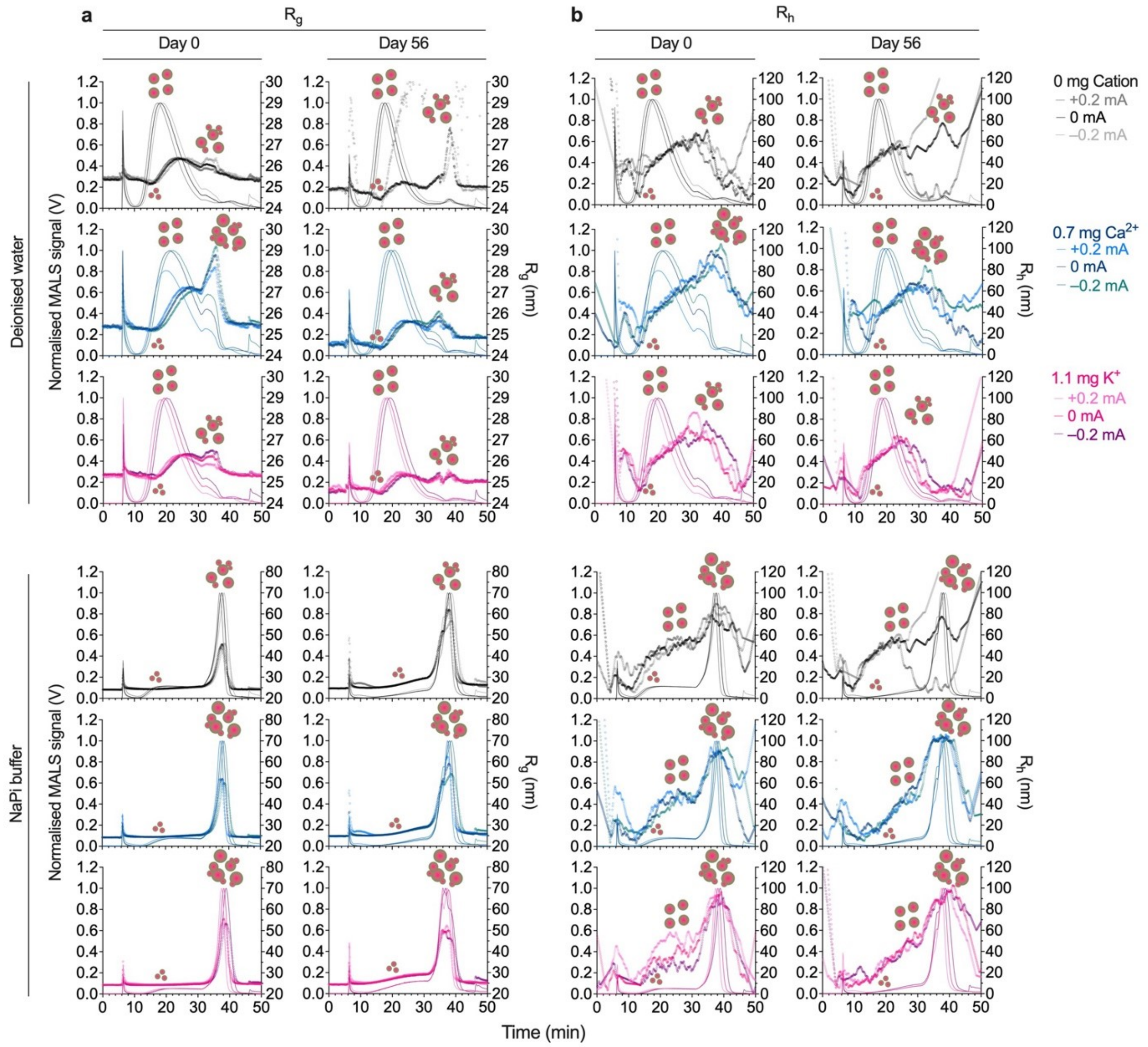

Fig. S4 Electrical asymmetric flow field-flow fractionation (EAF4) fractograms of silk nanoparticles (SNPs): 0 mg Cation SNP (black), 0.7 mg Ca^{2+} SNP (blue), and 1.1 mg K^{+} SNP (pink). The 0.1 mg/mL SNPs were separated in 0.5 mM sodium carbonate under electrical fields with applied positive (+0.2 mA), neutral (0 mA), and negative (–0.2 mA) currents. The SNP EAF4 fractograms for days 0 and 56 of storage in deionized water and 10 mM Sodium phosphate (NaPi) buffer (pH 7.4) at 4 °C were as a correlation of normalized MALS signal together with (**a**) radius of gyration (R_g) and **(b**) hydrodynamic radius (R_h) (n = 3).

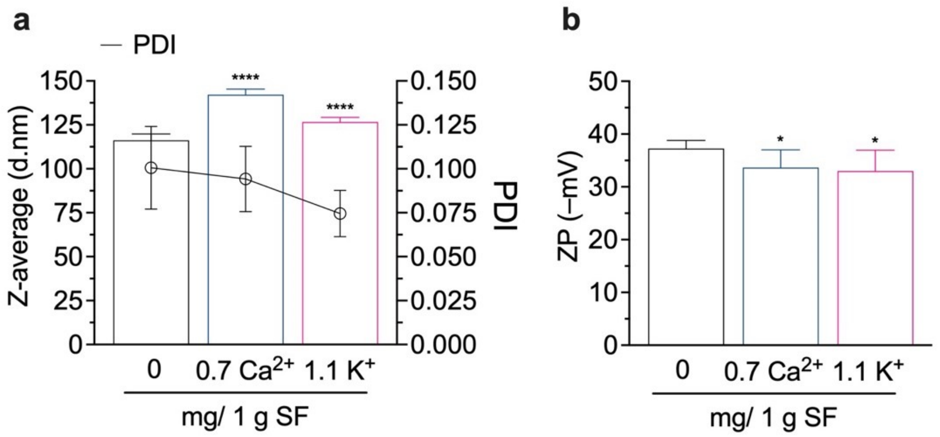

Fig. S5 Physicochemical properties of silk nanoparticles (SNPs) dispersed in 0.5 mM Na_2CO_3 for 1 h: (a) particle size and PDI analysis (dynamic light scattering; DLS); (b) ZP (electrophoretic light scattering; ELS). Two-way ANOVA, One-way ANOVA, and Dunnett's multiple comparisons test were assessed for statistical analyses comparable to 0 mg cation: $p < 0.05$ (*), $p < 0.01$ (**), $p < 0.001$ (***), and $p < 0.0001$ (****) (n = 3). Abbreviations: polydispersity index (PDI); zeta potential (ZP); silk fibroin (SF).

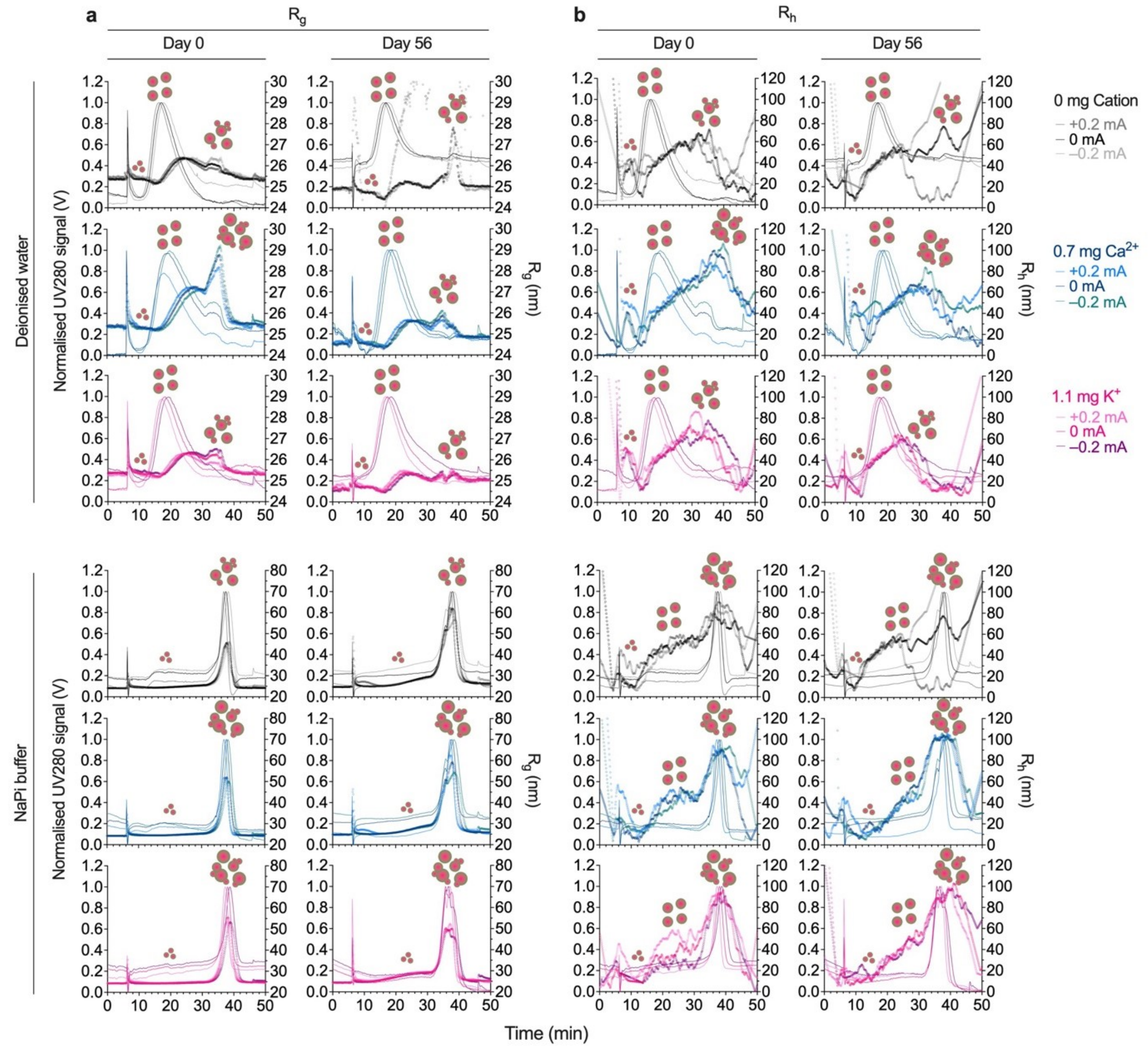

Fig. S6 Electrical asymmetric flow field-flow fractionation (EAF4) fractograms of silk nanoparticles (SNPs) : 0 mg Cation SNP (black), 0.7 mg Ca^{2+} SNP (blue), and 1.1 mg K^{+} SNP (pink). SNPs (0.1 mg/mL) were separated using a 0.5 mM sodium carbonate carrier liquid under with applied positive (+0.2 mA), neutral (0 mA), and negative (–0.2 mA) currents. The SNP EAF4 fractograms for days 0 and 56 of storage in deionized water and 10 mM Sodium phosphate (NaPi) buffer (pH 7.4) at 4 °C presented as a correlation of normalized UV 280 signal together with (**a**) radius of gyration (R_g) and **(b**) hydrodynamic radius (R_h) (n = 3).

Table S1 Silk nanoparticles (SNPs) dispersed in deionized (DI) water were derived from the electrical asymmetric flow field-flow fractionation (EAF4)-UV 280 detector. Two-way ANOVA and Šídák's multiple comparisons test were used in statistical analysis, comparing the impact of day 56 to day 0; $p < 0.05$ (*), $p < 0.01$ (**), $p < 0.001$ (***), and $p < 0.0001$ (****). Abbreviations: Peak 1 (P1); Peak 2 (P2); hydrodynamic radius (R_h); radius of gyration (R_g); dynamic light scattering (DLS); zeta potential (ZP).

SNP Sample	Applied current (mA)	Elution time (min)		MALS-R_g (nm)		DLS-R_h (nm)		R_g/R_h		Electrophoretic mobility (μm cm/V s)	Correlation coefficient (R^2)	ZP (mV)
		P1	P2	P1	P2	P1	P2	P1	P2			
**Day 0**												
0 mg Cation	+0.2	16.1 (±0.1)	32.8 (±0.5)	25.3 (±0.0)	25.9 (±0.0)	34.9 (±2.8)	53.8 (±15.8)	0.73 (±0.06)	0.51 (±0.14)	–4.68	0.9621	–59.91
	0	16.9 (±0.0)	32.7 (±0.2)	25.3 (±0.1)	26.1 (±0.0)	33.6 (±1.1)	67.1 (±19.9)	0.75 (±0.02)	0.42 (±0.41)			
	-0.2	17.8 (±0.2)	32.5 (±0.1)	25.3 (±0.1)	26.2 (±0.1)	37.6 (±4.6)	62.9 (±1.9)	0.68 (±0.09)	0.42 (±0.01)			
0.7 mg Ca2+	+0.2	18.0 (±0.2)	33.3 (±0.4)	25.6 (±0.1)	27.6 (±0.1)	42.1 (±3.7)	89.0 (±10.7)	0.61 (±0.06)	0.31 (±0.04)	–4.24	0.9696	–54.28
	0	18.9 (±0.1)	33.2 (±0.5)	25.6 (±0.1)	28.0 (±0.1)	40.7 (±1.5)	74.9 (±7.4)	0.63 (±0.02)	0.37 (±0.04)			
	-0.2	19.9 (±0.2)	33.5 (±0.5)	25.5 (±0.1)	28.1 (±0.2)	43.6 (±5.3)	68.6 (±8.3)	0.59 (±0.07)	0.41 (±0.05)			
1.1 mg K+	+0.2	17.0 (±0.2)	32.5 (±0.1)	25.3 (±0.1)	25.9 (±0.1)	39.1 (±0.5)	85.8 (±7.2)	0.65 (±0.01)	0.30 (±0.03)	–5.12	0.9845	–65.63
	0	17.9 (±0.4)	32.7 (±0.3)	25.3 (±0.1)	26.1 (±0.1)	35.1 (±3.0)	63.9 (±6.9)	0.73 (±0.06)	0.41 (±0.05)			
	-0.2	19.0 (±0.6)	32.8 (±0.1)	25.5 (±0.1)	26.4 (±0.0)	39.8 (±1.8)	64.6 (±15.3)	0.64 (±0.03)	0.42 (±0.10)			
**Day 56**												
0 mg Cation	+0.2	16.1 (±0.2)	37.9 (±0.2)****	23.5 (±1.9)***	28.3 (±0.1)*	42.2 (±0.9)**	5.5 (±0.7)****	0.56 (±0.06)***	5.23 (±0.73)****	–4.56	0.9621	–58.37
	0	16.9 (±0.1)	38.6 (±0.2)****	24.5 (±0.1)	27.6 (±0.1)	44.8 (±0.5)****	37.2 (±22.1)*	0.55 (±0.01)****	0.92 (±0.45)			
	-0.2	17.8 (±0.1)	39.5 (±0.1)****	24.7 (±0.1)	23.5 (±4.9)**	43.1 (±0.7)	103.9 (±24.3)***	0.57 (±0.01)*	0.24 (±0.10)			
0.7 mg Ca2+	+0.2	16.8 (±0.5)***	38.5 (±0.2)****	24.5 (±0.2)	25.2 (±0.1)*	49.0 (±2.2)*	19.8 (±5.9)****	0.50 (±0.02)*	1.34 (±0.35)	–5.01	0.9609	–64.17
	0	18.4 (±0.1)	33.4 (±0.0)	24.6 (±0.1)	25.8 (±0.1)*	49.0 (±2.0)**	29.5 (±3.3)****	0.50 (±0.02)**	0.88 (±0.09)			
	-0.2	19.5 (±0.3)	34.7 (±0.9)**	24.7 (±0.1)	26.0 (±0.1)*	49.4 (±1.7)*	35.8 (±4.2)**	0.50 (±0.02)	0.73 (±0.09)			
1.1 mg K+	+0.2	16.7 (±0.2)	33.3 (±0.8)	24.7 (±0.1)	25.1 (±0.1)	44.2 (±1.4)	2.9 (±0.4)****	0.56 (±0.02)	6.41 (±3.87)****	–4.86	0.8700	–62.28
	0	17.3 (±0.2)	33.2 (±0.3)	24.6 (±0.1)	25.1 (±0.1)	44.9 (±1.0)***	7.4 (±2.7)****	0.55 (±0.01)****	3.78 (±1.71)**			

	-0.2	18.4 (±0.4)*	33.3 (±0.1)	24.6 (±0.0)	25.2 (±0.0)	45.3 (±1.1)	13.4 (±6.3)****	0.54 (±0.01)*	2.17 (±0.96)

Table S2 Corresponding silk nanoparticles (SNPs) in 10 mM sodium phosphate (NaPi) buffer derived from electrical asymmetric flow field-flow fractionation (EAF4) -UV 280 detector. Two-way ANOVA and Šídák's multiple comparisons test were used in statistical analysis, comparing the impact of day 56 to day 0: $p < 0.05$ (*), $p < 0.01$ (**), $p < 0.001$ (***), and $p < 0.0001$ (****). Abbreviations: Peak 1 (P1); Peak 2 (P2); hydrodynamic radius (R_h); radius of gyration (R_g); dynamic light scattering (DLS); zeta potential (ZP); no data available (NA).

SNP Sample	Applied current (mA)	Elution time (min)		MALS-R_g (nm)		DLS-R_h (nm)		R_g/R_h	
		P1	P2	P1	P2	P1	P2	P1	P2
**Day 0**									
0 mg Cation	+0.2	14.7 (±0.3)	36.8 (±0.1)	24.5 (±0.1)	45.4 (±0.6)	33.7 (±2.6)	73.4 (±13.9)	0.73 (±0.06)	0.64 (±0.14)
	0	16.4 (±0.1)	37.4 (±0.1)	24.5 (±0.1)	44.7 (±1.4)	35.9 (±9.8)	79.1 (±3.3)	0.72 (±0.23)	0.58 (±0.03)
	-0.2	18.6 (±1.3)	38.3 (±0.1)	24.4 (±0.1)	45.6 (±0.4)	31.6 (±4.3)	76.4 (±16.4)	0.78 (±0.10)	0.62 (±0.15)
0.7 mg Ca2+	+0.2	18.9 (±2.7)	37.1 (±0.1)	24.6 (±0.1)	51.3 (±0.5)	38.0 (±5.3)	94.5 (±5.3)	0.66 (±0.09)	0.55 (±0.03)
	0	18.2 (±0.3)	37.8 (±0.1)	24.5 (±0.0)	51.7 (±0.3)	33.5 (±9.1)	81.8 (±12.8)	0.76 (±0.18)	0.64 (±0.09)
	-0.2	18.0 (±0.7)	38.4 (±0.0)	24.4 (±0.1)	49.7 (±0.3)	31.6 (±3.7)	87.5 (±4.3)	0.78 (±0.09)	0.57 (±0.03)
1.1 mg K+	+0.2	19.0 (±0.2)	37.3 (±0.0)	24.6 (±0.1)	57.5 (±0.8)	45.5 (±6.4)	91.1 (±6.0)	0.55 (±0.07)	0.63 (±0.04)
	0	19.3 (±0.4)	38.1 (±0.1)	24.3 (±0.0)	55.3 (±0.4)	32.9 (±3.4)	93.5 (±0.6)	0.74 (±0.08)	0.59 (±0.01)
	-0.2	19.1 (±0.1)	38.7 (±0.1)	24.3 (±0.1)	53.1 (±0.5)	26.4 (±4.4)	85.3 (±5.1)	0.94 (±0.15)	0.62 (±0.03)
**Day 56**									
0 mg Cation	+0.2	26.4 (±0.9)****	37.4 (±0.1)****	28.2 (±0.2)****	61.9 (±1.2)****	30.3 (±17.1)	11.8 (±1.6)****	1.12 (±0.52)	18.09 (±22.01)**
	0	30.3 (±0.2)****	38.0 (±0.1)****	29.5 (±0.2)****	61.8 (±0.2)****	56.0 (±12.6)	78.3 (±42.5)	0.55 (±0.14)	1.17 (±0.20)
	-0.2	30.4 (±0.2)****	38.5 (±0.1)***	29.4 (±0.1)****	56.5 (±0.1)****	74.2 (±39.0)**	197.1 (±30.0)****	0.53 (±0.40)	1.37 (±1.87)
0.7 mg Ca2+	+0.2	27.1 (±0.8)****	37.4 (±0.0)****	28.2 (±0.1)****	62.4 (±1.5)****	43.8 (±6.3)	102.9 (±2.5)	0.65 (±0.09)	0.61 (±0.02)
	0	26.6 (±0.0)****	37.9 (±0.1)	28.0 (±0.2)****	58.9 (±0.6)****	48.3 (±19.9)	103.6 (±9.5)	0.64 (±0.21)	0.57 (±0.05)
	-0.2	26.6 (±0.0)****	38.6 (±0.1)*	27.8 (±0.1)****	54.2 (±0.4)****	39.7 (±9.9)	100.6 (±6.5)	0.73 (±0.17)	0.54 (±0.03)

	+0.2	28.0 (±1.0)****	37.2 (±0.0)	29.4 (±0.2)****	51.9 (±0.6)****	44.1(±7.1)	103.1(±1.5)	0.68 (±0.11)	0.50 (±0.01)
1.1 mg K+	0	27.6 (±1.3)****	37.7 (±0.0)****	29.0 (±0.2)****	49.9 (±0.3)****	56.8 (±20.1)	103.2 (±7.9)	0.56 (±0.20)	0.49 (±0.03)
	-0.2	29.7 (±0.5)****	38.2 (±0.1)****	29.0 (±0.1)****	46.1 (±0.2)****	44.5 (±7.4)	102.1 (±5.1)	0.66 (±0.11)	0.45 (±0.02)
